# Bioinspired Tissue Transparency: Achieving Sclera‐to‐Cornea Transplantation

**DOI:** 10.1002/advs.202514871

**Published:** 2026-01-04

**Authors:** Xiuli Sun, Long Zhao, Zhen Shi, Jingting Wang, Shang Yang, Xia Qi, Hengrui Zhang, Ting Wang, Weiyun Shi

**Affiliations:** ^1^ Department of Ophthalmology Affiliated Hospital of Shandong Second Medical University Weifang P. R. China; ^2^ State Key Laboratory Cultivation Base Shandong Key Laboratory of Eye Diseases Eye Institute of Shandong First Medical University School of Ophthalmology Shandong First Medical University Qingdao P. R. China; ^3^ No. One Clinical Medicine School Binzhou Medical University Binzhou P. R. China; ^4^ Eye Institute of Shandong First Medical University Eye Hospital of Shandong First Medical University (Shandong Eye Hospital) Jinan P. R. China

**Keywords:** allogeneic transplantation, bioinspired tissue clearing, corneal substitute, tissue engineering

## Abstract

Human soft tissues have significant clinical potential for corneal repair, yet their therapeutic efficacy is limited by inherent opacity. This study introduces a bio‐inspired decellularization‐compression‐locking tactic (DCLT) for tissue clearing. The DCLT leads to long‐term transparency (>80% light transmittance) of biological soft tissues while preserving native bioactivity by removing light‐scattering subcellular structures and regulating extracellular matrix fiber density. This distinguishes it from traditional reagent‐mediated tissue‐clearing approaches. Using DCLT, transparent decellularized human sclera (hTDS) is fabricated as a corneal substitute, integrating advantages of optical clarity, mechanical reinforcement, and resistance to swelling and enzymatic degradation. hTDS promotes wound healing and restores optical function in models of complex corneal injuries, including alkali burns, acute edematous‐phase keratoconus, and open‐globe injuries. Notably, its anti‐angiogenic and anti‐fibrotic efficacy are superior to those of donor corneas in ocular surface with chronic inflammation. This study establishes a simple, effective, and safe tissue‐clearing strategy and provides a promising tissue engineering solution for allogeneic corneal transplantation.

## Graphical abstract

A bioinspired decellularization‐compression‐locking tactic (DCLT) is developed to transform human sclerae into transparent corneal substitutes. These clear, robust, and pro‐regenerative substitutes are capable of repairing complex corneal injuries, including chemical burns, keratoconus, and penetrating wounds, demonstrating their clinical potential to address corneal blindness and donor shortage.

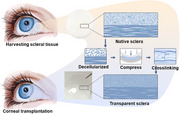

## 1 Introduction

Corneal blindness affects more than 10 million individuals globally and poses a significant public health challenge. Corneal transplantation is the gold standard for vision restoration. However, donor scarcity severely constrains it, resulting in an annual transplantation rate of less than 1.5% [1]. This substantial therapeutic gap has prompted extensive research on corneal substitutes, focusing on three primary material classes: xenogeneic biomaterial scaffolds (e.g., acellular cornea, bio‐derived hydrogels), optical prostheses (keratoprosthesis), and human soft tissues (e.g., sclera, derma, and conjunctiva) [2]. Despite promising clinical potential, each approach has limitations. The xenogeneic bioscaffolds remain experimental, facing hurdles like inherent rejection risks, susceptibility to melting in inflammatory microenvironments, and structural fragility [3, 4, 5, 6]. Fully synthetic keratoprostheses offer greater stability, but their limited biointegration often leads to serious complications, such as retroprosthetic membrane and corneal melting [7, 8]. In contrast, human soft tissues have been the most clinically utilized corneal substitutes for over a century, due to their immunocompatibility, mechanical matching, and high availability [9, 10, 11, 12, 13]. These materials generally improve prognosis and facilitate patient acceptance. However, their significant optical mismatch with the cornea often leads to suboptimal visual recovery [9, 13, 14]. Consequently, developing transparent human tissues for corneal repair represents an attractive but underexplored approach.

Tissue opacity primarily arises from microstructural heterogeneities that attenuate light [15]. Extracellularly, complex matrix fibrils cause scattering due to refractive index (RI) mismatches between the hydrated macromolecules (*n'* ≈ 1.44) and interstitial fluid (*n'* ≈ 1.35) [16]. Intracellularly, organelles and protein aggregates induce additional Mie scattering because their sizes match the visible wavelengths. Endogenous pigments (such as heme) also block light transmission [17]. Existing tissue‐clearing methods primarily focus on optical imaging of biotissues, such as CLARITY and 3DISCO [18]. They involve replacing tissue water with high‐RI agents or removing most biocomponents to create an all‐aqueous environment [19, 20]. These are effective for enhancing tissue transparency; however, their application to in vivo transplantation faces two key challenges: dependency on cytotoxic agents (e.g., tetrahydrofuran [21], acrylamide [22]) and re‐opacification upon clearing‐agent removal [15]. These limitations highlight the clinical need for advanced tissue‐clearing strategies that ensure biocompatibility and long‐term transparency.

The exceptional transparency of the cornea is attributed to the tight arrangement of the extracellular matrix (ECM) with inter‐fibrillar spacings of approximately 20 nm maintained by lamellar pressure, along with a sparse population of quiescent stromal cells interspersed among the fibers (Figure S1) [23]. Transparency diminishes as a result of extensive infiltration of opaque myofibroblasts and increased ECM fiber spacing caused by corneal injury or edema (Figure S2) [24]. Based on these findings, we hypothesized that removing light‐scattering cells and increasing matrix density would improve tissue transparency.

**FIGURE 1:**
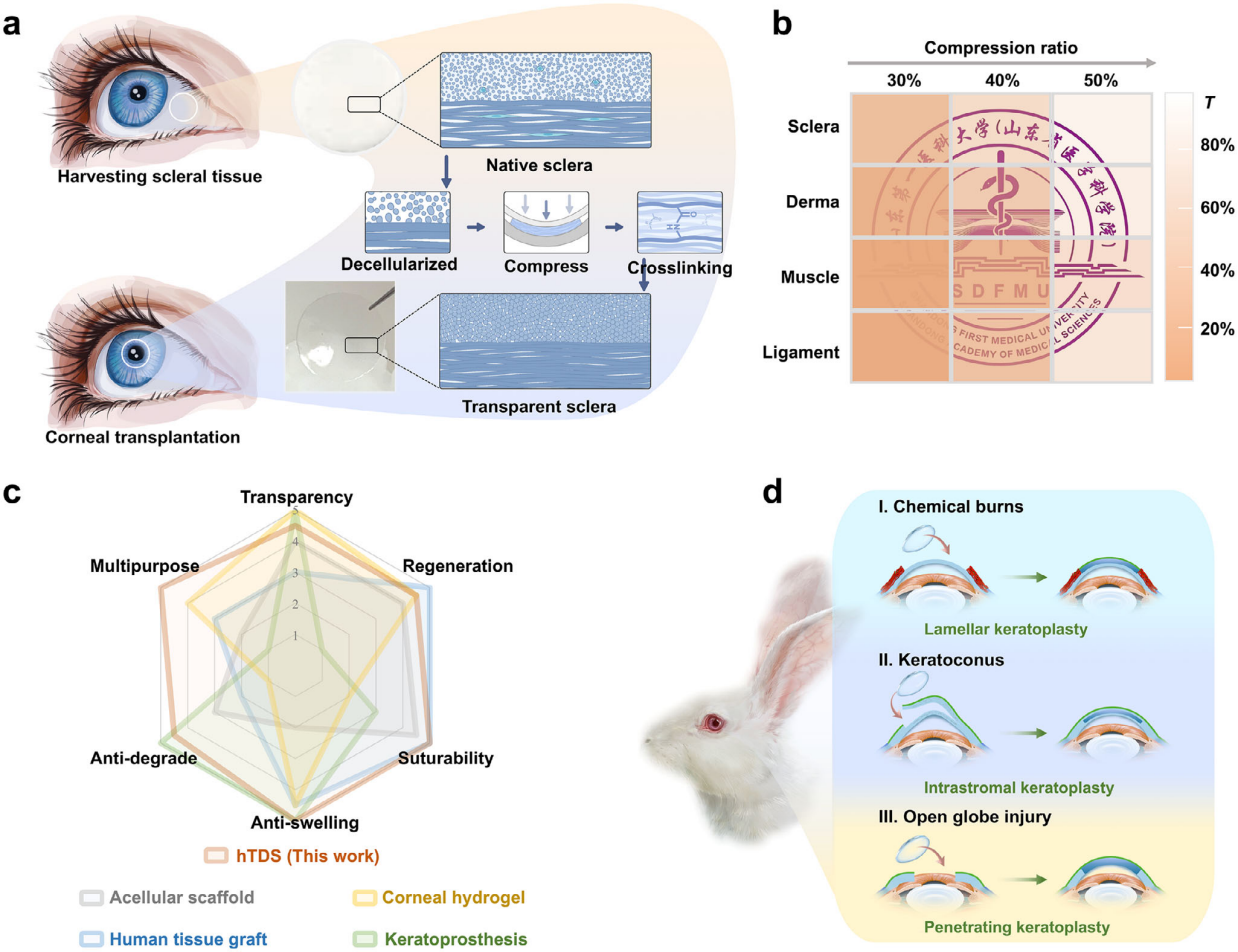
Preparation workflow and performance advantages of transparentized tissue by DCLT. (a) Schematic illustration of the transformation from native sclera to transparentized sclera for corneal transplantation. (b) Transparency variations in sclera, derma, muscle, and ligament tissues under 30%–50% compression ratios at 550 nm wavelength. The logo of Shandong First Medical University is used with permission. (c) The hTDS integrates key functional advantages, with each parameter approaching or exceeding those of current clinical and experimental corneal substitutes, including acellular scaffold, corneal hydrogel, human tissue graft, and keratoprosthesis, as qualitatively shown in a radar chart. Indexes: (1 = poor; 5 = outstanding). The appraisal standards are detailed in Table S1. (d) Potential applications of hTDS in challenging corneal injury models: lamellar keratoplasty for chemical burns, intrastromal keratoplasty for keratoconus, and penetrating keratoplasty for open globe injury.

The cornea‐inspired decellularization‐compression‐locking tactic (DCLT) was developed for tissue clearing. First, cellular components are removed through decellularization to eliminate light scattering from subcellular structures and mitigate immunogenicity. Subsequently, controlled compression induces matrix densification and RI homogenization by reducing inter‐fibrillar spacing. Finally, chemical crosslinking “locks” the architecture by forming covalent bonds between adjacent fibrils (Figure 1a; Figure S3). DCLT achieves tunable transparency by adjusting compression intensity and is broadly applicable to biological soft tissues such as the sclera, ligament, derma, and muscle (Figure 1b).

The sclera and cornea together constitute the outer wall of the eyeball, exhibiting structural similarity and continuity. Furthermore, the sclera has an area 5.5 times larger than the cornea and a longer preservation time, resulting in relatively abundant eye bank reserves [25]. Therefore, the human sclera was selected as a transplant candidate. The transparent decellularized human sclera (hTDS) prepared using DCLT meets key criteria for corneal substitutes, including optical transparency (>80% transmittance at 550 nm), proregenerative capacity (epithelial healing >92.5% at 14 d), suture resistance (9.1 MPa tensile strength), hydration stability (water uptake <5%), enzymatic resistance (94.2% retention after 5d collagenase), and abundant human ECM (162 ECM‐related proteins; Figure 1c). These features support its evaluation in three challenging ocular surface trauma models: (I) chemical burns with neovascularization and chronic inflammation, (II) acute hydrops in advanced keratoconus requiring urgent mechanical reinforcement, and (III) open globe injury with full‐thickness corneal defect (Figure 1d).

## 2 Results and Discussion

### 2.1 Validation of Transparency Mechanism

**FIGURE 2:**
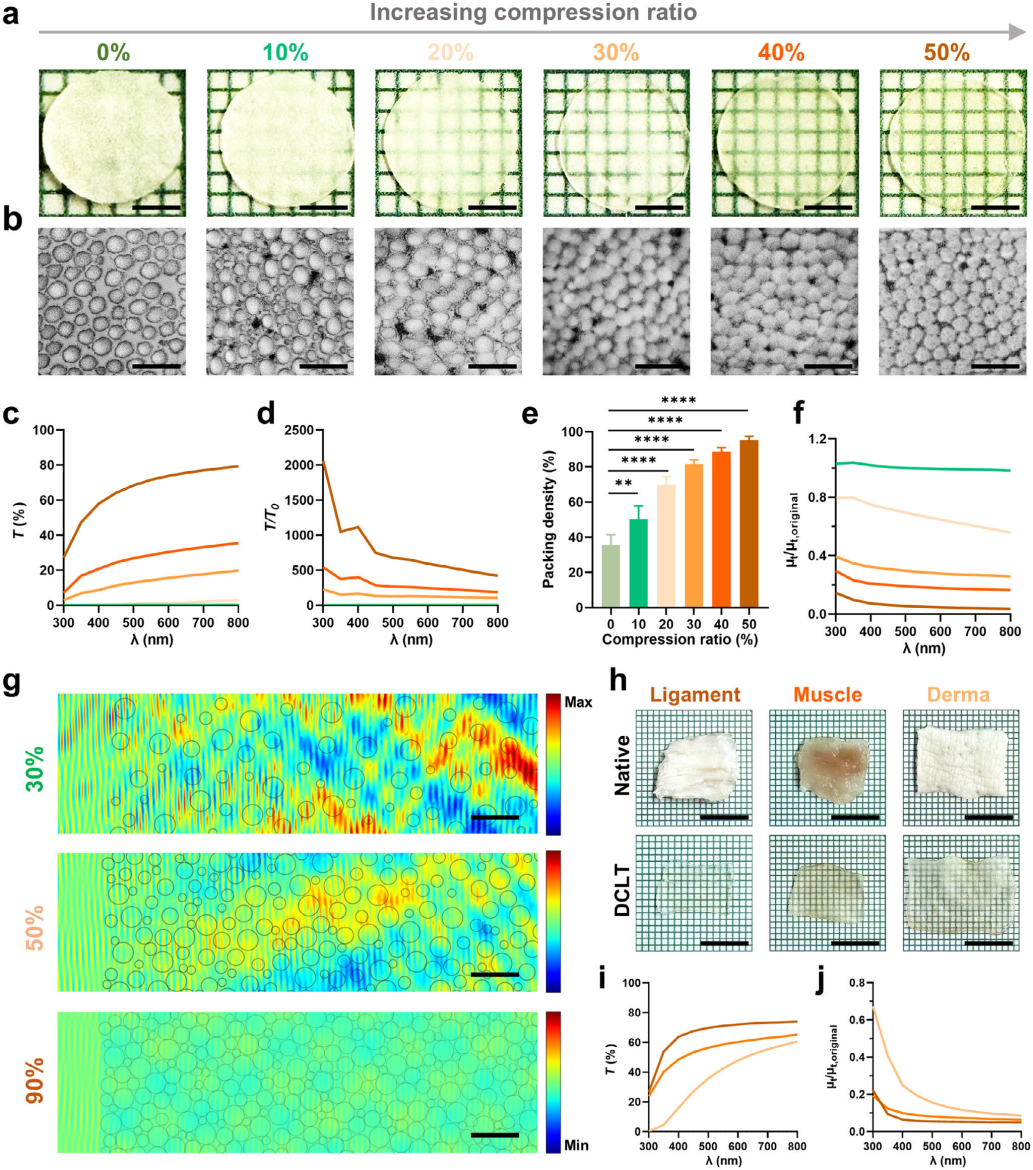
Validation of tissue optical clearing mechanism. (a) Macroscopic images depicting the transition of decellularized sclerae from opacity to transparency under increasing compression ratios (0%–50%). Scale bar: 3 mm. (b) Transmission electron microscopy (TEM) analysis revealing progressive matrix densification. Scale bar: 350 nm. (c) Light transmittance spectra of decellularized sclerae at various compression ratios. (d) Transmission enhancement factor spectra, defined as the transmittance ratio of compressed to uncompressed sclera. (e) Matrix fiber packing density at varying compression ratios (*n* = 4 independent samples; ANOVA followed by Tukey's multiple comparisons; ** adjusted *p* = 0.003, **** adjusted *p* < 0.0001; data are presented as mean ± SD). (f) Attenuation coefficient ratio of compressed decellularized sclera relative to the original tissue. Color‐coded in panels c–f corresponds to the compression ratio labels in panel a. (g) Numerical simulation of plane wave propagation through aqueous matrices containing scattering particles with packing densities of 30%, 50%, and 90%. Scale bars: 500 nm. (h) Macroscopic comparison between native and DCLT‐treated ligament, muscle, and dermal tissues. Scale bar: 12 mm. (i) Light transmittance spectra and (j) attenuation coefficient reduction ratios of DCLT‐treated tissues, with color coding corresponding to tissue types in panel h.

The scleral tissues were decellularized using the methods we previously established to eliminate cellular components that cause light attenuation [26, 27]. The decellularized sclera was subjected to controlled mechanical compression and positioned on a millimeter‐gridded substrate. Bright‐field imaging revealed that compression triggered a progressive transition from opacity to transparency (Figure 2a). Quantitative analysis of normal‐incidence light transmittance (*T*) confirmed a compression‐dependent optical enhancement (Figure 2c). The sclera had corneal‐comparable transmittance at 50% compression and nearing ultimate strain, with *T* increasing by more than 600‐fold within the visual sensitivity range (500–550 nm; Figure 2d).

This optical transformation originates from changes in the density of matrix fibril packing. The native scleral matrix had a low packing density (35.6 ± 5.9%) and wide interfibrillar gaps (79.5 ± 30.6 nm) filled with interstitial fluid, leading to an RI mismatch (Figure 2e; Figure S1f). Mechanical compression reduced these distances to 7.68 ± 4.1 nm and increased the fibril packing density to 95.2 ± 2.2% at 50% compression (Figure 2e). High‐density packing led to a nonlinear increase in the interfacial contact ratio (Figure S4). Direct fibril‐fibril contact eliminated the collagen‐fluid interfaces and minimized RI contrast (Δ*n'* ≈ 0), as confirmed by a reduced normalized attenuation coefficient (Figure 2f). Additionally, the formation of a percolation‐like contact network promoted local hexagonal ordering (Figure 2b). This geometric ordering enhances forward photon transport efficiency by minimizing optical path deviation. The optical observations were supported by finite‐difference time‐domain (FDTD) simulations: the incident planar waves had waveform deformation for low‐density fibril arrangements, whereas their propagation was nearly undisturbed at a 90% packing density (Figure 2g).

Tissue clearing induced by mechanical compression was reversible upon hydration within minutes (Figure S5a,b). Chemical crosslinking via carbodiimide‐mediated amide bond formation between adjacent fibrils was employed to “lock” the remodeled ultrastructural configuration for the preservation of optical transparency (Figure S5c–g). In addition, DCLT successfully enhanced transparency in ligament, muscle, and derma (Figure 2h). Its role as a universal mechanism for optical clearing in soft tissues has been validated (Figure 2i,j; Figure S6).

### 2.2 Biophysical Characteristics of hTDS

**FIGURE 3:**
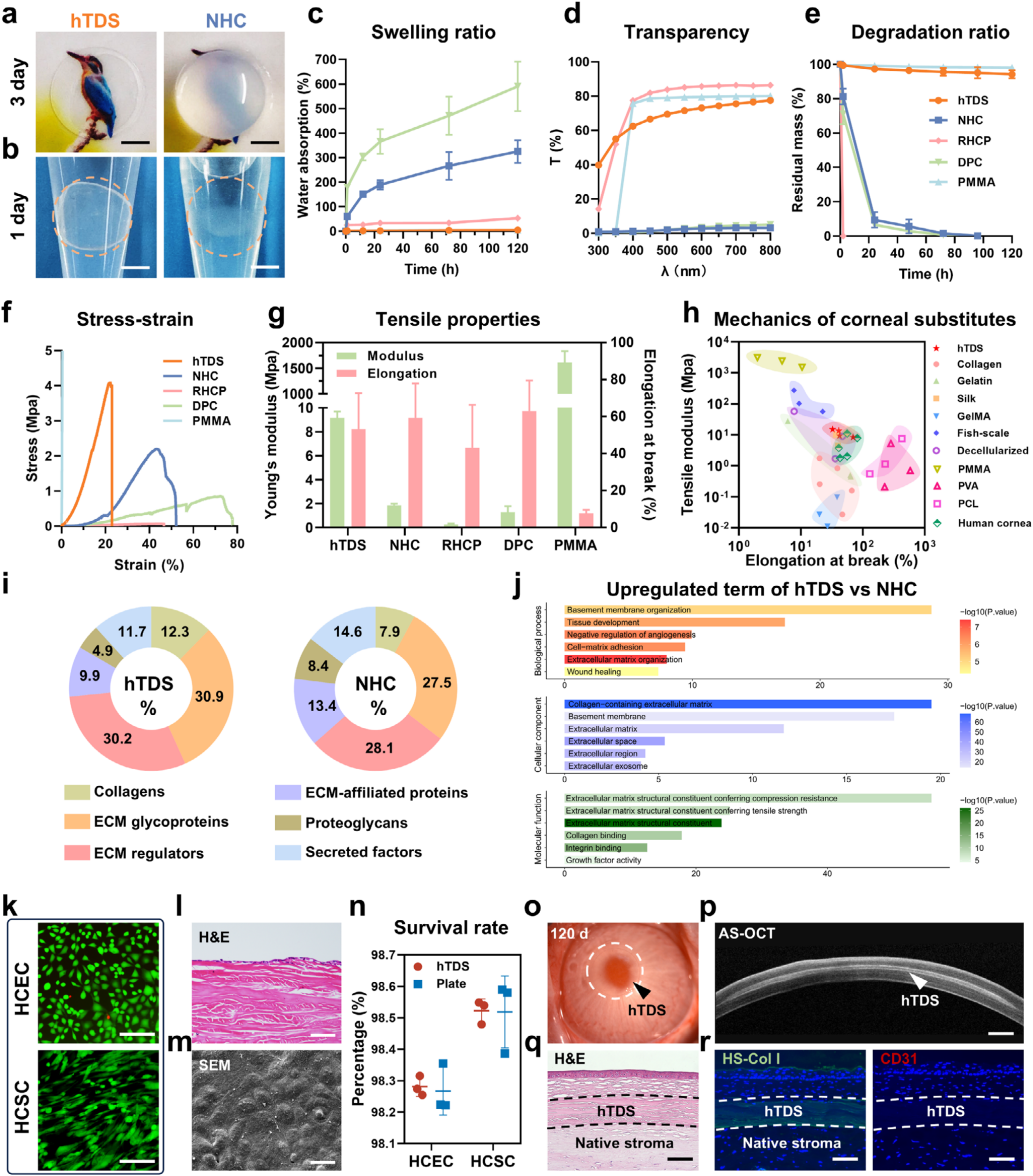
Biophysical characteristics and biocompatibility of hTDS. (a) Macroscopic comparison of hTDS and NHC after a three‐day immersion in artificial tear solution. Scale bar: 3 mm. (b) Structural integrity of hTDS versus NHC after 24‐h collagenase treatment. Scale bar: 3.5 mm. (c) Water absorption kinetics of hTDS, NHC, and representative corneal substitutes (RHCP, DPC, PMMA) over 120 h (n = 3 independent samples; data are presented as mean ± SD). (d) Representative transmittance spectra after 5‐day hydration. (e) Residual mass percentage following collagenase digestion (n = 4 independent samples; data are presented as mean ± SD). Curves in panel c–e correspond to the color scheme in panel e. (f) Representative tensile stress‐strain curves. (g) Young's modulus and elongation at break of tested materials (n = 3 independent samples; data are presented as mean ± SD). (h) Ashby plot comparing mechanical properties of hTDS with those of human cornea [30, 35] and literature‐reported substitutes: synthetic polymers (PMMA [36], PVA [37, 38, 39], and PCL [40]), biologically derived materials (decellularized corneas [41, 42], silk fibroin [43, 44, 45], and fish‐scale derivatives [46, 47, 48]), and collagen‐based scaffolds (collagen [49, 50], gelatin [51, 52], and GelMA [53, 54, 55]). (i) Matrisome protein subtype distribution in hTDS and NHC. (j) Significantly upregulated GO terms in hTDS relative to NHC. (k) Representative live/dead fluorescence staining images of HCECs and HCSCs. Scale bar: 150 µm. (l) H&E staining demonstrates the formation of a multilayered epithelium by HCECs on hTDS. Scale bar: 50 µm. (m) SEM image of HCECs showing intercellular junctions. Scale bar: 10 µm. (n) Survival rate of HCECs and HCSCs (n = 3 independent samples; Student's *t* test; two‐tailed *p* = 0.651, and 0.968 for HCECs and HCSCs, respectively; data are presented as mean ± SD). (o) Bright field and (p) AS‐OCT images of hTDS implants in rabbit cornea at 120 d. Scale bar: 500 µm. (q) H&E staining of hTDS integrating with corneal stroma at 120 d. Scale bar: 100 µm. (r) Immunofluorescence localization of HS‐Col I (green) and vascular endothelium marker CD31 (red) at 120 d post‐implantation. Scale bar: 100 µm.

The water absorption and optical performance of hTDS were evaluated via immersion in artificial tears. The controls included the native human cornea (NHC), decellularized porcine cornea (DPC), recombinant human collagen patch (RHCP), and PMMA (the optical component of the Boston keratoprosthesis). All have been used in clinical practice or trials for corneal substitution. The hTDS maintained dimensional stability and optical clarity after immersion for 3 days, whereas NHC exhibited significant edema (Figure 3a; Figure S7). Quantitative analysis revealed that hTDS had minimal water absorption rate (4.69 ± 1.51%) over 5 days, outperforming DPC (325.19 ± 45.83%) and NHC (590.57 ± 101.03%; Figure 3c). hTDS retained more than 70% light transmittance at 500–800 nm post‐immersion, whereas NHC and DPC retained less than 5% (Figure 3d).

Graft melting is a major cause of corneal transplantation failure [28]. The degradation resistance of hTDS was evaluated using a 50 U mL^−1^ high collagenase activity assay. hTDS maintained its original shape after 24 h of incubation at 37°C, whereas NHC showed partial melting with blurred boundaries (Figure 3b; Figure S8). Quantitative analysis showed hTDS retained 94.2 ± 2.3% of the residual mass after 5 days. In contrast, other collagen‐based materials had near‐complete degradation (Figure 3e).

### 2.3 Biomechanical Properties of hTDS

Biomechanical compatibility is essential for corneal substitutes to integrate with host tissue and resist pathological deformation [29]. The uniaxial tensile testing of hTDS revealed a J‐shaped stress‐strain curve, which is characterized by an initial low‐modulus phase (0%–5% strain) followed by a strain‐stiffening phase due to the recruitment of crimped collagen fibrils to bear load. This is consistent with the viscoelasticity of the native cornea (Figure 3f; Figure S9a) [30]. Quantitative analysis revealed that the hTDS had a Young's modulus of 8.41 ± 0.83 MPa, which was 33 times higher than that of RHCP (0.25 ± 0.05 MPa) and threefold greater than that of NHC (2.47 ± 0.54 MPa). The hTDS also maintained comparable ductility (elongation at break: 46.1 ± 16.3%) to that of the native cornea (Figure 3g). Stress relaxation tests indicated that the stress retention of hTDS was superior to those of NHC and DPC under equivalent strain (Figure S9b,c). As shown in Figure 3h, compared with most natural and synthetic material‐derived substitutes, the hTDS exhibited balanced stiffness and stretchability, similar to the mechanical properties of human corneas [31].

### 2.4 Compositional Characterization of hTDS

The matrisome database, an ensemble of genes encoding ECM‐associated proteins, was used to compare the protein composition of hTDS and NHC [32]. hTDS exhibited matrisome subtypes closely resembling those of the human cornea, including 20 types of collagens, 50 glycoproteins, and 8 proteoglycans (Figure 3i; Figure S10a). Notably, hTDS contains 40% more collagen isoforms than NHC, potentially explaining its enhanced mechanical performance (Figure 3g) [33]. Conversely, hTDS demonstrated a 47% reduction in proteoglycan diversity compared with NHC, which likely contributed to its superior dimensional stability in aqueous environments due to the known hygroscopicity of proteoglycans [34].

Differential expression analysis identified 252 upregulated and 156 downregulated proteins in hTDS versus NHC (Figure S10b,c). Gene ontology (GO) enrichment analysis of the upregulated proteins revealed three key functional clusters in hTDS. Molecular function annotation revealed significant enrichment for “ECM structural constituents conferring tensile strength and compression resistance,” corroborating its favorable mechanical properties. Cellular component mapping revealed predominant localization to the “basement membrane” and “collagen‐containing ECM”, underscoring its role in epithelial adhesion and structural support. Biological process analysis revealed upregulated pathways such as “wound healing” and “tissue development”, demonstrating its potential for corneal regeneration (Figure 3j).

### 2.5 Biocompatibility of hTDS

The multiscale biocompatibility assessment was conducted. The human corneal epithelial cells (HCECs) and stromal cells (HCSCs) cultured with hTDS‐conditioned medium had over 98% viability, as revealed by live/dead staining (Figure 3k,n). Quantitative analysis confirmed the normal proliferation kinetics of corneal cells, with consistent increases in cell density (Figure S11). The in vitro three‐dimensional (3D) culture of hTDS resulted in the formation of a confluent 2–3 layered epithelium on its surface within one week (Figure 3l). Scanning electron microscopy (SEM) confirmed the establishment of intercellular tight junctions (Figure 3m).

The hTDS was implanted into the rabbit cornea for in vivo biocompatibility evaluation. It maintained optical transparency comparable to that of the recipient cornea after 120 days, as shown by slit‐lamp microscopy (Figure 3o). The implant‐host integration was confirmed by anterior segment optical coherence tomography (AS‐OCT), without interfacial fluid accumulation (Figure 3p). Hematoxylin and eosin (H&E) staining revealed no fibroblast encapsulation or inflammatory cell infiltration around hTDS (Figure 3q). The structural integrity of hTDS was confirmed by immunofluorescence staining using the human‐specific collagen I (HS‐Col I) antibody. CD31‐negative staining indicated the absence of neovascularization at the implantation site (Figure 3r). These findings highlight the immunological inertness and structural durability of hTDS, positioning it as a promising allogeneic implant for corneal tissue engineering.

### 2.6 In Vivo Lamellar Stromal Repair With hTDS

To evaluate the clinical viability of hTDS, a rabbit lamellar keratoplasty model (6.0‐mm diameter, 200‐µm depth) was established (Figure S12a). The hTDS‐transplanted corneas successfully achieved epithelial healing and maintained transparency over the 28‐day follow‐up, as confirmed by slit‐lamp biomicroscopy (Figure S12b,e,f). In contrast, the untreated group had progressive corneal scarring (Figure S12c). Pachymetry maps confirmed that the central thicknesses of hTDS‐repaired corneas (423.5 ± 9.8 µm) closely matched those of the allograft group (415.2 ± 8.3 µm; Figure S12d,g).

**FIGURE 4:**
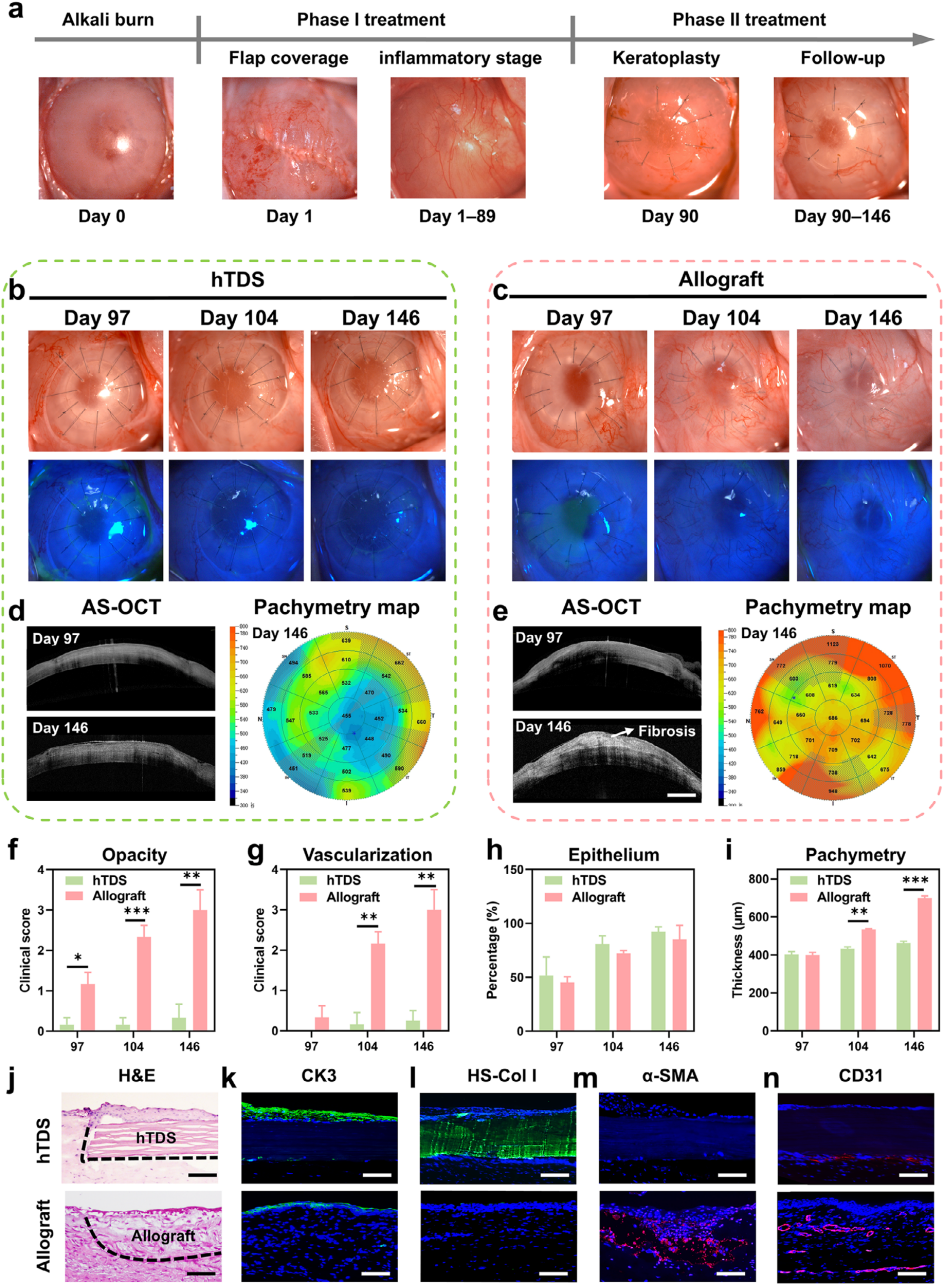
Therapeutic efficacy of hTDS versus allografts in alkali‐burned corneas. (a) Timeline of model establishment and a two‐stage therapeutic protocol: Phase I involving autologous conjunctival flap transplantation to mitigate inflammation and prevent infection, followed by Phase II corneal transplantation for optical functionality restoration. (b) Representative bright‐field and fluorescence staining images of hTDS and (c) allograft groups. (d) AS‐OCT images and pachymetry maps of hTDS and (e) allograft groups. Scale bars: 1 mm. The AS‐OCT data were obtained from longitudinal follow‐up of the same rabbit. (f) Progression of corneal opacity (n=4 independent samples; Student's *t* test; two‐tailed *p* = 0.013 and 0.004 for 97 and 146 days, *p*<0.001 for 104 day, respectively; data are presented as mean ± SD). (g) Progression of neovascularization (n=4 independent samples; Student's *t* test; two‐tailed *p* = 0.116, 0.001, and 0.001 for 97, 104, and 146 days, respectively; data are presented as mean ± SD). (h) Epithelial healing rate (n=4 independent samples; Student's *t* test; two‐tailed *p* = 0.547, 0.143, and 0.407 for 97, 104, and 146 days, respectively; data are presented as mean ± SD). (i) Central corneal thickness progression (n=4 independent samples; Student's *t* test; two‐tailed *p* = 0.874 for 97 day, *P*<0.0001 for 104 and 146 days, respectively; data are presented as mean ± SD). (j) Representative H&E staining images at 146‐d endpoint. Scale bars: 100 µm. (k) Immunofluorescence staining for CK3, (l) HS‐Col I, (m) α‐SMA, and (n) CD31. Scale bars: 100 µm.

Nearly 50% of corneal transplantation involve compromised ocular surface microenvironments characterized by chronic inflammation. Conventional keratoplasty has suboptimal outcomes and frequent complications, including neovascularization and graft melting under these conditions [56, 57]. To model such conditions, we developed a full‐corneal alkali burn model, and adopted a two‐stage therapeutic strategy consistent with surgical consensus [58]. The stages were as follows: (I) acute inflammatory phase (1–89 days post‐injury): necrotic tissue debridement and conjunctival flap coverage were performed to minimize infection risks and facilitate resolution of inflammation; and (II) reconstructive phase (90–146 days): the central optical zone was reconstructed using 6.25‐mm hTDS transplantation, with allogeneic corneal grafts serving as controls (Figure 4a; Figure S13).

After 56 days of follow‐up, hTDS maintained graft transparency with minimal inflammation (Figure 4b,f). Compared to allografts, the hTDS group achieved accelerated re‐epithelialization and suppressed fibrovascular membrane formation more effectively (Figure 4b,c,g,h). AS‐OCT imaging further confirmed the restoration of normal corneal architecture in the hTDS group, which was characterized by homogeneous stromal density and near‐physiological thickness (463.3 ± 9.2 µm). In contrast, the allograft‐treated corneas had fibrous membrane coverage and pathological thickening (698.5 ± 12.1 µm) at 146 days (Figures 4e,i).

H&E staining of the hTDS‐repaired corneas revealed no immune cell infiltration and fibroblast hyperproliferation at the graft edge at 146 days (Figure 4j). Immunofluorescence analysis revealed a well‐structured multilayered epithelium on hTDS with robust expression of the terminal differentiation marker CK3, indicating functional epithelial restoration (Figure 4k). HS‐Col I staining confirmed seamless integration of hTDS with the recipient bed (Figure 4l). The hTDS‐repaired corneas had minimal α‐SMA expression, suggesting limited myofibroblast activation (Figure 4m). CD31 expression was significantly lower in the hTDS‐repaired corneas than in the allograft controls, confirming the inhibition of neovascularization (Figure 4n). These findings demonstrate that hTDS effectively repairs pathological lamellar defects with minimal fibrotic remodeling.

### 2.7 In Vivo Repair of Acute Edematous‐phase Keratoconus Using hTDS

**FIGURE 5:**
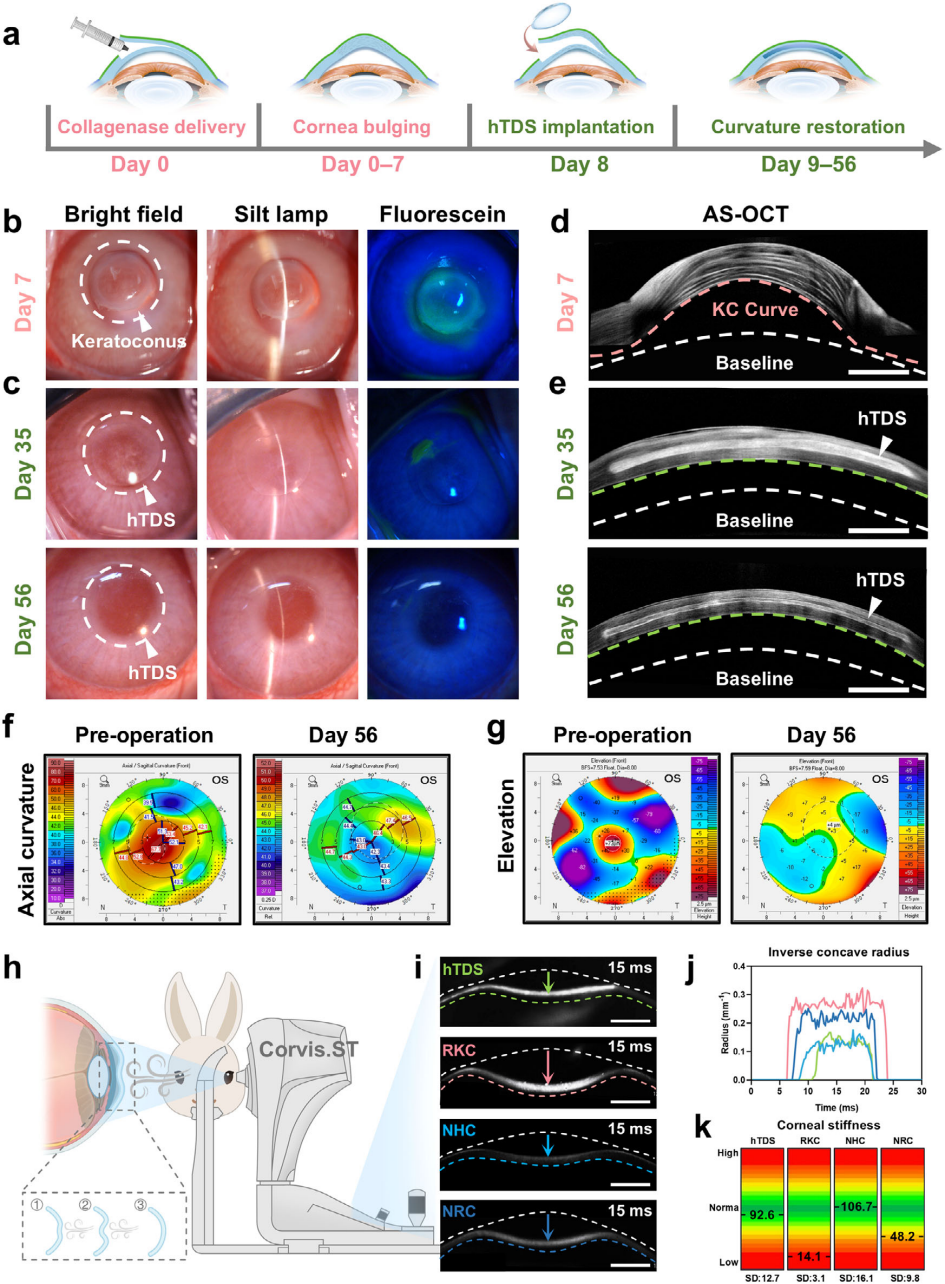
Therapeutic evaluation of hTDS for acute edematous keratoconus. (a) Schematic timeline of keratoconus model establishment and hTDS implantation. The model was established over days 0‐7, followed by hTDS implantation on day 8, and a treatment period of days 9–56. (b) Preoperative and (c) postoperative representative bright‐field, slit‐lamp, and fluorescein images of keratoconus corneas. (d) Preoperative and (e) postoperative AS‐OCT images. KC curve: keratoconus curvature; green curve: curvature after hTDS treatment; white curve: baseline curvature. Scale bars: 1 mm. Corneal topography maps show (f) axial curvature and (g) anterior elevation pre‐ and post‐hTDS treatment. (h) Schematic of Corvis ST biomechanical assessment: ① Air‐puff induced corneal deformation; ② Dynamic corneal response; and ③ Stiffness parameter derivation. (i) Post‐air‐puff AS‐OCT images of hTDS‐implanted, rabbit keratoconus (RKC), native rabbit cornea (NRC) and NHC. White curves: baseline profile; colored curves: corneal profile at 15 ms; arrows: apex‐to‐concavity trajectory. Scale bars: 1 mm. (j) Representative inverse concave radius variations across groups, color matched to panel g. (k) Corneal stiffness distribution in hTDS, RKC, NHC, and NRC (n=4 independent samples; data are presented as mean). Green: normal range; red: pathological range.

Keratoconus is a progressive ectatic corneal disorder characterized by collagenase overexpression, stromal thinning, and biomechanical instability. The aqueous humor infiltrates the corneal stroma and epithelium during the acute hydrops stage, leading to rapid vision deterioration and eventual blindness [59]. To induce pathological corneal thinning, collagenase (0.1 U mL^−1^) was topically applied to the rabbit stromal pocket (Figure 5a). The corneas exhibited typical features of keratoconus, including central bulging, reduced transparency, and extensive epithelial detachment resulting from stromal edema and eyelid friction, on day 7 after enzyme treatment (Figure 5b). Rapid edema resolution, progressive restoration of transparency, and re‐epithelialization were observed after hTDS interlamellar implantation (Figure 5c; Figure S14). The delayed epithelial healing on day 35 was likely due to the steep corneal surface and tear film instability, which impeded epithelial migration [60]. AS‐OCT imaging confirmed the reversal of acute interlamellar separation and significant reduction in corneal protrusion, which approached the baseline curvature after hTDS intervention (Figure 5d,e). Histological staining revealed infiltration of surrounding stromal cells into the graft, indicating integration of the hTDS with the adjacent corneal stroma (Figure S15).

Pentacam topography revealed significant improvement in anterior corneal topography. The maximum keratometry (Kmax) decreased from 49.2 ± 7.6 D preoperatively to 44.5 ± 1.5 D post‐hTDS implantation, aligning with the healthy range (44.6 ± 1.4; Figure 5f) [61]. Elevation mapping further confirmed morphological restoration. The diseased cornea displayed a characteristic “island‐shaped” central protrusion (Δ = 66.2 ± 11.7 µm) before treatment, which decreased to 1.7 ± 3.2 µm after hTDS treatment (Figure 5g). Corneal biomechanics were assessed using the Corvis tonometer. The cornea was subjected to an air‐puff impulse, and its deformation was recorded using an ultra‐high‐speed camera (Figure 5h). The hTDS‐implanted corneas had smaller deformations than the untreated control, and the inverse concave radius curve closely matched those of the normal human corneas (Figure 5i,j). The stiffness parameter at the first applanation (SP‐A1), which reflects corneal resistance to deformation under external pressure, increased from 14.1 ± 3.1 to 92.6 ± 12.7 and was within the human corneal physiological range (Figure 5k). These findings indicate that hTDS interlamellar implantation facilitates the restoration of corneal morphology and biomechanical functionality and demonstrates its therapeutic potential for acute edematous‐phase keratoconus.

### 2.8 hTDS for In Vivo Sealing of Full‐Thickness Corneal Defects

**FIGURE 6:**
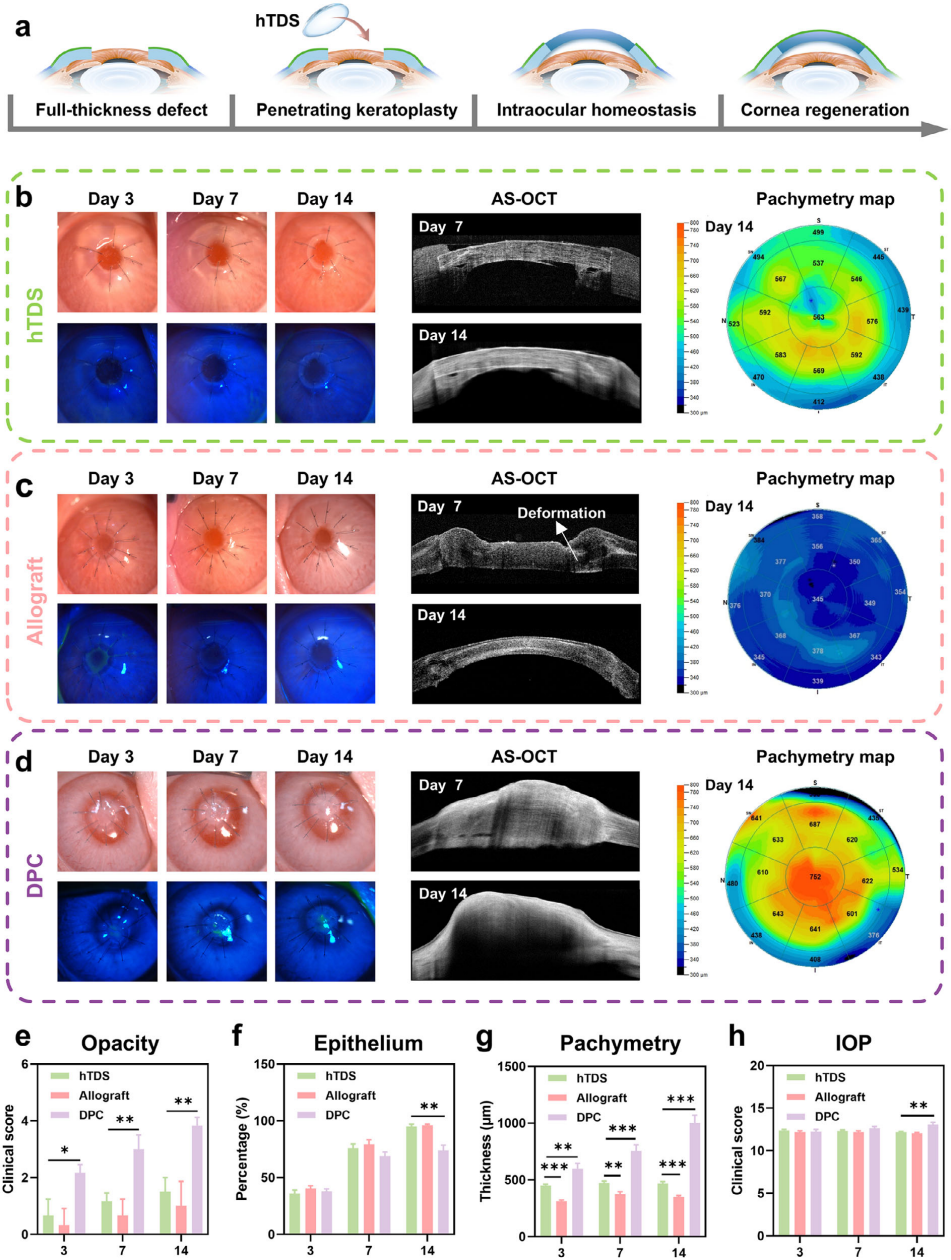
Comparative evaluation of hTDS, allografts, and DPC in repairing full‐thickness corneal defect. (a) Schematic of open globe injury model establishment and hTDS administration. (b) Representative bright‐field, fluorescein staining, AS‐OCT, and pachymetry maps of hTDS‐treated, (c) allograft‐treated, and (d) DPC‐treated corneas. Scale bars: 1 mm. (e) Progression of corneal opacity in hTDS, allograft, and DPC groups. (f) Epithelial healing percentage. (g) Central corneal thickness progression and (h) intraocular pressure (IOP) in different treatment groups. e–h: n=4 independent samples; ANOVA followed by Tukey's multiple comparisons; * adjusted *p* < 0.05, ** adjusted *p* < 0.01, *** adjusted *p* < 0.001; data are presented as mean ± SD.

A 4.0‐mm‐diameter penetrating corneal defect model was established to simulate open globe injuries (Figure 6a). Slit‐lamp and AS‐OCT imaging revealed anterior chamber collapse and anterior displacement of intraocular contents (Figure S16). The sealing efficacy was evaluated using hTDS grafts with a diameter of 4.25 mm secured with interrupted 10–0 nylon sutures (6–7 stitches). The hTDS group required nearly 50% fewer sutures than conventional keratoplasty (Figure 6b,c), due to its superior mechanical support from the high‐modulus hTDS (Figure 3g).

The hTDS maintained optical clarity during the follow‐up period, and all treated eyes had restored anterior chamber depth without aqueous leakage (Figure 6b,e). Complete epithelialization was achieved in the hTDS‐treated corneas by two weeks post‐implantation, with no fluorescein staining (Figure 6b,f). AS‐OCT imaging demonstrated good graft‐host integration with minimal edema and natural curvature (Figure 6b,g). In contrast, the allograft controls exhibited localized graft deformation due to suture traction (Figure 6c). The DPC group developed marked corneal edema and opacification (thickness >1000 µm at 14 d; Figures 6d,g). The intraocular pressure in the hTDS‐treated corneas stabilized at approximately 12 mmHg, which is within the normal range (10–20 mmHg; Figure 6h). The restoration of both anatomical integrity and physiological function supports hTDS as an effective emergency intervention for full‐thickness corneal defects.

## 3 Conclusion

This study presents DCLT as the only technology to date that leverages synergistic physicochemical regulation to enhance the optical performance of biotissues. This simple, low‐cost approach enhances the light transmittance of tissues by at least two orders of magnitude while retaining native bioactivity, meeting the requirements for in vivo transplantation. The hTDS prepared by DCLT has several advantages over conventional corneal substitutes: 1) superior structural stability that resists graft melting and swelling in compromised ocular microenvironments; 2) robust yet ductile mechanical properties that provide structural support for pathologically thinned corneas; and 3) species‐matched genetic identity and abundant ECM that enhance immunocompatibility and regeneration. The hTDS demonstrated significant repair efficacy across multiple challenging pathological models, including stromal defects, alkali burns, keratoconus, and open globe injuries, outperforming donor corneas in suppressing fibrosis and neovascularization. This constitutes the first comprehensive validation of a bioengineered corneal substitute in such complex scenarios. The DCLT pioneers a novel paradigm for fabricating transparent human corneal substitutes and holds promise for addressing multiple ocular surface challenges.

## 4 Experimental Section

### 4.1 Materials and Sources

SuperNuclease was purchased from Sino Biological (Beijing, China). Collagenase, 4‐(4,6‐dimethoxy–1,3,5‐triazin‐2‐yl)‐4‐methylmorpholinium chloride (DMTMM), and sodium lauroylglutamate were purchased from Sigma‐Aldrich (MO, USA). 1‐Ethyl‐3‐(3‐dimethylaminopropyl) carbodiimide (EDC) and N‐hydroxysuccinimide (NHS) were acquired from Macklin (Shanghai, China). Recombinant human collagen was obtained from Jinbo (Shanxi, China). 1× phosphate‐buffered saline (PBS), 4% paraformaldehyde, and 3% bovine serum albumin were purchased from SolarBio (Beijing, China). The live/dead double‐staining kit was obtained from Abbkine (GA, USA). Fetal bovine serum was purchased from Hyclone (UT, USA). The reagents for TEM and SEM were procured from Head Biotechnology (Beijing, China).

The human cornea and sclera tissues were preserved at the Eye Bank of the Eye Hospital of Shandong First Medical University. The Ethics Committee of Shandong Eye Hospital approved the tissue collection (approval no. SDSYKYY202204‐1). Voluntary informed consent was obtained from the donors or their immediate family members. Fresh porcine corneas, ligaments, skin, and muscle tissues were purchased from local suppliers and stored frozen.

The Simian virus 40‐immortalized HCECs (ATCC, CRL‐11135, Manassas, VA) were cultured in Dulbecco's modified Eagle's medium (DMEM)/F12 (Sigma, St. Louis, MO) supplemented with 10% fetal bovine serum at 37°C and 5% CO_2_. The telomerase‐immortalized HCSCs were maintained under identical conditions as provided by Dr. Jester [62].

### 4.2 Tissue‐Clearing by DCLT

The glycerol‐preserved human sclerae were rehydrated in PBS until their native tissue hydration status was restored for the preparation of hTDS. The posterior pole specimens were extracted using 10 mm diameter trephine burs and then embedded in optimal cutting temperature (O.C.T.) compound (Tissue‐Tek, SAKURA, USA) and snap‐frozen at –80°C. Cryo‐sectioning was performed using a cryostat microtome (CM3050S, Leica, Germany) to produce 300‐µm slices for lamellar and intrastromal keratoplasty and 600‐µm slices for in vitro assays and full‐thickness transplantation. Decellularization involved immersion in 2% (v/v) Triton X‐100 for 4 h at room temperature, followed by enzymatic treatment with 2000 U mL^−1^ SuperNuclease combined with 2% Triton X‐100 at 37°C for 12 h. Thorough washing was performed using PBS through six consecutive 1‐h cycles.

The decellularized sclerae were compressed using a manual flattening machine (Durston, Desborough, UK), with the compression gap controlled by a high‐precision digital caliper. The decellularized sclerae were placed in customized corneal contact lens molds (43.0–44.0D, Lylap, China) for in vivo implantation specimens and compressed to achieve a predefined curvature. This compression molding process was maintained for 72 h at 25°C and 50% relative humidity. The scaffolds were immersed in a 3% (w/v) DMTMM solution (pH 6.5) for 30 min to induce covalent cross‐linking (Figure S17). They were subsequently washed three times with PBS. Terminal sterilization was achieved through combined glow discharge plasma treatment (DK6640 system, Wuhan, China) and gamma irradiation (12 kGy dose from a ^60^Co‐γ source). All procedures were performed in a Class 100 cleanroom. The porcine ligaments, dermis, and skeletal muscle tissues underwent similar processing: thawing at ambient temperature for 5 min, horizontal sectioning into 2–5 mm slices, and decellularization, compression, and cross‐linking, as described above.

### 4.3 Macroscopic Transparency and Light Transmittance

The macroscopic transparency was evaluated by photographing the samples positioned on standardized printed substrates (1.5 × 1.5 mm grid) under white light‐emitting diode panel illumination. The spectral absorbance of the samples (*n* = 3) in the 300–800 nm range was measured using an ultraviolet‐visible spectrophotometer (Molecular Devices, Silicon Valley, CA) after they were placed in quartz cuvettes. Data analysis was performed using SoftMax Pro version 4.8 (Molecular Devices). The attenuation coefficient ratio of compressed tissue (*µ_t_*) relative to the original tissue (*µ_t,original_*) was calculated using Equation (1):

(1) $$\frac{\mu_{t}}{\mu_{t,original}}=\frac{\ln T_{original}}{\ln T_{compress}}$$

### 4.4 Numerical Simulation of Light Propagation

The simulations were performed using the FDTD method. The computational domain (2 × 7 µm^2^) comprised two components: randomly distributed collagen fiber scatterers (50–250 nm diameter) and background interstitial fluid medium. The transverse electric polarized plane wave source (λ = 550 nm) was incident from the top boundary under periodic lateral boundary conditions. The matrix fiber volume packing density was systematically varied from 30% to 90% to simulate different compression states. The RIs were set to 1.47 for the matrix fibers and 1.35 for the interstitial fluid. The temporal resolution was controlled by a Courant stability factor of 0.99, and the total simulation duration was 9000 fs to ensure field stabilization. Perfectly matched layer boundary conditions were implemented to minimize nonphysical reflections along the propagation direction.

### 4.5 Preparation of Control Materials

The RHCP was prepared following an established protocol [50, 63]. The 13.7% (w/v) recombinant human collagen solution was homogenized with EDC and NHS at molar ratios of 0.5:0.5:1 (EDC: NHS: collagen–NH2). The mixture was immediately dispensed into contact lens molds and crosslinked at 100% humidity and 21°C for 24 h, followed by 37°C for another 24 h. The materials were thoroughly washed three times with PBS for 10 min each.

The DPC was prepared according to the reported protocols [64]. The epithelium‐free anterior corneal stroma (thickness: 400 µm; diameter: 9 mm) was excised using a femtosecond laser‐cutting machine (Wavelight FS200, Fort Worth, TX). The corneal lenticules underwent dual‐cycle high hydrostatic pressurization (200 MPa, 2 min) and immersion in decellularization buffer containing 0.5% sodium lauroylglutamate and 500 U mL^−1^ SuperNuclease. This process was maintained at 37°C with a colloid osmotic pressure of 50 mmHg for 2 h. The final samples were washed thoroughly with sterile PBS.

### 4.6 Histological Staining

H&E staining was performed using commercial kits (Vector Laboratories, Burlingame, CA) according to the manufacturer's protocols. The samples (*n* = 3) were cryo‐embedded in O.C.T. and sectioned into 7 µm slices using a cryostat for immunofluorescence. The slides were fixed with 4% paraformaldehyde for 15 min and washed with PBS. The samples were blocked with 3% bovine serum albumin for 1 h at room temperature and incubated with primary antibodies (detailed in Table S2) overnight at 4°C in a humidified chamber. The sections were washed with PBS and incubated with secondary antibodies for 1 h at room temperature. The slides were mounted with ProLong Gold Antifade Mountant containing DAPI (Thermo Fisher Scientific, Waltham, MA) and sealed with coverslips after washing three times with PBS. Fluorescent images were acquired using a confocal laser scanning microscope (LSM 880, Carl Zeiss, Oberkochen, Germany) and analyzed using ZEN Lite software (Version 3.8).

### 4.7 Ultrastructural Analysis

For TEM, samples (*n* = 3) were fixed in 2.5% (v/v) glutaraldehyde (4°C, 4 h) and 1% osmium tetroxide (4°C, 2 h). They were then dehydrated with graded acetone, embedded in Epon812 resin, and cured for 24 h at 60°C. Ultrathin sections (60 nm) were prepared using an ultramicrotome (UC6, Leica, Wetzlar, Germany) and contrast‐stained with 2% (w/v) aqueous uranyl acetate for 15 min and 1% (w/v) aqueous lead citrate for 10 min. The electron microscope (JEM‐1400, JEOL, Tokyo, Japan) was used for observation, and images were captured with a digital camera (iTEM 5.0, Olympus, Tokyo, Japan). The fiber packing density, minimum interfibrillar distance, and fiber diameter, were measured via ImageJ (National Institutes of Health, Bethesda, MD) based on established methods [65]. Fiber packing density was defined as the percentage of the total area in a TEM micrograph occupied by matrix fibers. For SEM analysis, the specimens fixed with glutaraldehyde were subjected to ethanol dehydration, critical point drying, and 5‐nm gold sputter coating. The microstructure was evaluated using SEM (VEGA3, TESCAN, Brno, Czech) at an accelerating voltage of 15 kV.

### 4.8 Swelling Behavior Analysis

The swelling kinetics were characterized by monitoring mass changes in triplicate samples. The initial sample weights (*W_0_*) were recorded using a microbalance (Mettler Toledo, Greifensee, Switzerland). The specimens were immersed in artificial tears (Table S3) at 37°C for 5 days and weighed daily at predetermined time points (*W_t_*). The swelling ratio was calculated using Equation (2).

(2)

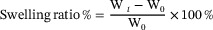




### 4.9 Enzyme Resistance Assay

The samples (n = 3) were incubated in a collagenase solution (50 U mL^−1^) prepared in PBS (pH 7.4) at 37°C for 5 days after their initial weights (*W_0_*) were recorded. They were weighed at predetermined times (*W_t_*), and the residual mass percentage was calculated using Equation (3).

(3) $$\mathrm{Residual}\;\mathrm{mass}\;\%=\frac{W_{t}}{W_{0}}\times 100\;\%$$

### 4.10 Mechanical Measurements

Tensile testing was performed using a universal testing machine (AGS‐X‐10KN, Shimadzu, Kyoto, Japan) at a stretching speed of 1 mm min^−1^. Strain was calculated as the ratio of the change in length to the initial length, and stress was calculated by dividing the force by the cross‐sectional area of the sample. Young's modulus was estimated from the slope of the initial linear region of the stress‐strain curve. The samples were pre‐stretched to 120% of their initial length at a speed of 0.4 mm s^−1^ for 300 s while recording force decay for stress relaxation analysis.

### 4.11 Proteomic Analysis

The three frozen tissue specimens were cryogenically pulverized in liquid nitrogen. The protein lysates were subsequently incubated on ice for 30 min and sonicated for 2 min. The supernatants were collected after centrifugation at 4°C for 20 min, and the protein concentrations were quantified using the bicinchoninic acid assay. Equal amounts of protein were digested with trypsin according to standard protocols. Peptide separation was performed using a nanoflow system (EASY‐nLC 1200, Thermo Scientific, Waltham, MA) coupled with a timsTOF Pro mass spectrometer (Bruker Daltonics, Billerica, MA) equipped with an Evosep One ion source. Chromatographic separation was performed on a reversed‐phase C18 column (25 cm × 75 µm) using a 90‐min linear gradient. The MS/MS data were processed using MaxQuant (v2.1.3.0) against the Homo sapiens UniProt database (2023.02). The proteins with |log_2_ FC| > 1 and *p*‐value < 0.05 were subjected to GO enrichment analysis.

### 4.12 Cytocompatibility Assessment

HCECs and HCSCs were seeded into 12‐well plates (1 × 10^5^ cells per well) and allowed to adhere for 12 h. The hTDSs were pre‐conditioned in complete medium for 24 h to generate leachates, which were subsequently used to replace the culture medium for a 36‐h exposure. The control groups received fresh serum‐supplemented medium. Cell viability was assessed using a live/dead double dye kit and visualized using a phase‐contrast microscope (Nikon Eclipse Ti‐U, Tokyo, Japan). The living cells were quantified using Photoshop CS (Adobe, San Jose, CA). Cell proliferation was monitored daily using the Cell Counting Kit‐8 (CCK‐8, Dojindo, Japan) according to the manufacturer's instructions. Three replicates were used for each condition.

### 4.13 In Vitro 3D Culture

The hTDSs were anchored to 12‐well plates using 1% (w/v) agar. HCECs (1 × 10^5^) were then seeded onto the material surfaces and cultured in complete medium for 7 days. Specimens were collected for histological analysis after culture. The samples (n = 3) were fixed in 10% neutral buffered formalin for 24 h, processed through graded alcohols, embedded in paraffin, and cut into 4 µm sections. The sections were stained with H&E following standard protocols.

### 4.14 Experimental Animals and Anesthesia

Healthy male New Zealand White rabbits (aged 5–6 months; Xilingjiao, Jinan, China) were used in this study. All animal experiments strictly adhered to the Association for Research in Vision and Ophthalmology Statement for the Use of Animals in Ophthalmic and Vision Research and were approved by the Ethics Committee of the Eye Institute of Shandong First Medical University (Approval No. SDSYKYJS 20230415). Surgical anesthesia was induced via intramuscular injection of ketamine (40 mg kg^−1^) and chlorpromazine (20 mg kg^−1^) and supplemented with topical 0.5% proparacaine hydrochloride (Alcon, Puurs, Belgium).

### 4.15 Alkali Burn Model

A 12‐mm filter saturated with 1 M NaOH was applied to the cornea for 40 s after anesthesia. This was followed by immediate pulsed irrigation with 0.9% sterile saline for 5 min in the conjunctival sac. Perilimbal conjunctival peritomy was subsequently performed, followed by debridement of necrotic tissue and dissection of Tenon's capsule. The superior and inferior conjunctival flaps were transposed and secured with continuous 10–0 nylon sutures to ensure complete corneal coverage. Postoperative care included topical erythromycin ointment (Bausch & Lomb, Bridgewater, NJ) applied twice daily until suture removal on postoperative day 7.

### 4.16 Keratoconus Model

A midstromal pocket was created using a modified lamellar dissection technique after anesthesia. A circular mark with a diameter of 3.5 mm was made on the corneal surface using a trephine. A 1‐mm lamellar incision was made at the marked edge, followed by blunt dissection parallel to the corneal surface using a 27‐gauge iris repositor. A 30‐gauge cannula connected to a microsyringe was inserted into the pocket to inject 10 µL of collagenase type I solution (1.5 mg mL^−1^). The enzymatic reaction was allowed to proceed for 20 min, followed by pulsatile irrigation of physiological saline through the incision. The postoperative care included the application of erythromycin ointment twice daily for 7 days.

For the surgical treatment of keratoconus, intrastromal keratoplasty was performed as follows: A circular mark was created on the central cornea using a 6.5 mm trephine. An arcuate incision was made along the marked site, and a corneal lamellar dissector was used to create a 6.5 mm stromal cavity. The hTDS implant (6.0 mm in diameter, 60–80 µm in thickness) was carefully inserted into the pocket using micro forceps, and residual fluid or gas bubbles were removed using an iris repositor. The silicone hydrogel bandage contact lens was applied postoperatively, and 0.3% tobramycin/0.1% dexamethasone ophthalmic ointment (s.a. Alcon‐Couvreur n.v., Puurs, Belgium) was administered topically twice daily for 7 days.

### 4.17 Lamellar Keratoplasty

Central stromal circular incisions were created in the normal and alkali‐burned corneas to a depth of 200–250 µm using a 6.0 mm surgical trephine under AS‐OCT guidance. Recipient beds were subsequently created using a lamellar knife. The hTDS implants (6.25 mm diameter) were secured to the stromal beds using 10–0 nylon sutures. The control groups included allogeneic rabbit corneal transplantation using the same surgical procedure as the hTDS group, and untreated defects that underwent lamellar keratectomy but did not receive a reparative implant (*n* = 4 per group). Postoperative care included twice‐daily application of tobramycin and dexamethasone ophthalmic ointment for the first 5 days, followed by an equivalent eye drop formulation until postoperative day 14.

### 4.18 Penetrating Keratoplasty

Circular full‐thickness corneal defects (4.0 mm in diameter) were created in the central corneas using a surgical trephine. The wound edges were adjusted with micro‐scissors to achieve a perpendicular incision geometry. The hTDS implants (4.25 mm diameter) were secured using 6–7 interrupted 10–0 nylon sutures. The control groups received either allogeneic rabbit corneas or DPC grafts (n = 4 per group). Postoperative therapy followed the lamellar keratoplasty protocol.

### 4.19 Clinical Assessments

Corneal clarity, neovascularization, and inflammatory responses were evaluated using slit lamp microscopy (SL‐D701, Topcon, Tokyo, Japan). The integrity of the corneal epithelium was assessed using fluorescein sodium staining, and the areas of the epithelial defects (fluorescein‐stained regions) were quantified using ImageJ software. Corneal opacity and vascularization was graded according to a semiquantitative preclinical ocular toxicology scoring (SPOTS) system (Table S4) [66]. AS‐OCT was used to capture cross‐sectional images and measure central corneal thickness. The IOP values were measured using a rebound tonometer (TonoVet, West Jordan, UT). Corneal topographic was conducted using the Pentacam anterior segment analysis system (OCULUS, Wetzlar, Germany). The corneal biomechanical properties were characterized using the Corvis ST noncontact tonometer (OCULUS, Wetzlar, Germany).

### 4.20 Statistical Analysis

Statistical analyses were performed using GraphPad Prism software (version 8.3.0, GraphPad, San Diego, CA). The data are presented as means ± standard deviations. The data of two groups were compared using the Student's t‐test. The data of more than two groups were analyzed using one‐way analysis of variance (ANOVA) and Tukey's post‐hoc test. The sample size (n) for each test is described in this section. Each experiment was performed independently at least three times. All tests were two‐sided. Statistical significance was set at *p* < 0.05.

## Conflicts of Interest

The authors declare no conflicts of interest.

## Supporting information




**Supporting File**: advs73467‐sup‐0001‐SuppMat.docx.

## Data Availability

The data that support the findings of this study are available from the corresponding author upon reasonable request.
