# Supplementary material for: Bioinspired Tissue Transparency: Achieving Sclera‐to‐Cornea Transplantation
Source: Adv Sci (Weinh). 2026 Jan 4;13(10):e14871. doi: 10.1002/advs.202514871 (PMC12915115; doi:10.1002/advs.202514871)
Supplement: Supplementary file 1 — Supporting File: advs73467‐sup‐0001‐SuppMat.docx. [file ADVS-13-e14871-s001.docx]

**Supplementary Information**

**Bioinspired Tissue Transparency: Achieving Sclera-to-Cornea Transplantation**

*Xiuli Sun, Long Zhao*, Zhen Shi, Jingting Wang, Shang Yang, Xia Qi, Hengrui Zhang, Ting Wang*, Weiyun Shi**

L. Zhao, Z. Shi, J. T. Wang, X. Qi, H. R. Zhang, W. Y. Shi

Eye Institute of Shandong First Medical University, State Key Laboratory Cultivation Base, Shandong Key Laboratory of Eye Diseases, School of Ophthalmology, Shandong First Medical University, Qingdao 266071, P. R. China.

*Corresponding authors

E-mail: wyshi@sdfmu.edu.cn (W. Y. Shi); zhaolong@email.sdfmu.edu.cn (L. Zhao)

X. L. Sun

Department of Ophthalmology, Affiliated hospital of Shandong Second Medical University, Weifang 261053, China

S. Yang

Binzhou Medical University, Binzhou 264003, China

T. Wang

Eye Institute of Shandong First Medical University, Eye Hospital of Shandong First Medical University (Shandong Eye Hospital), Jinan 250021, China

E-mail: [wting@sdfmu.edu.cn](mailto:wting@sdfmu.edu.cnm) (T. Wang)

This file includes:

Figure S1 to S17

Table S1 to S4

Reference

**1. Supplementary Figures**


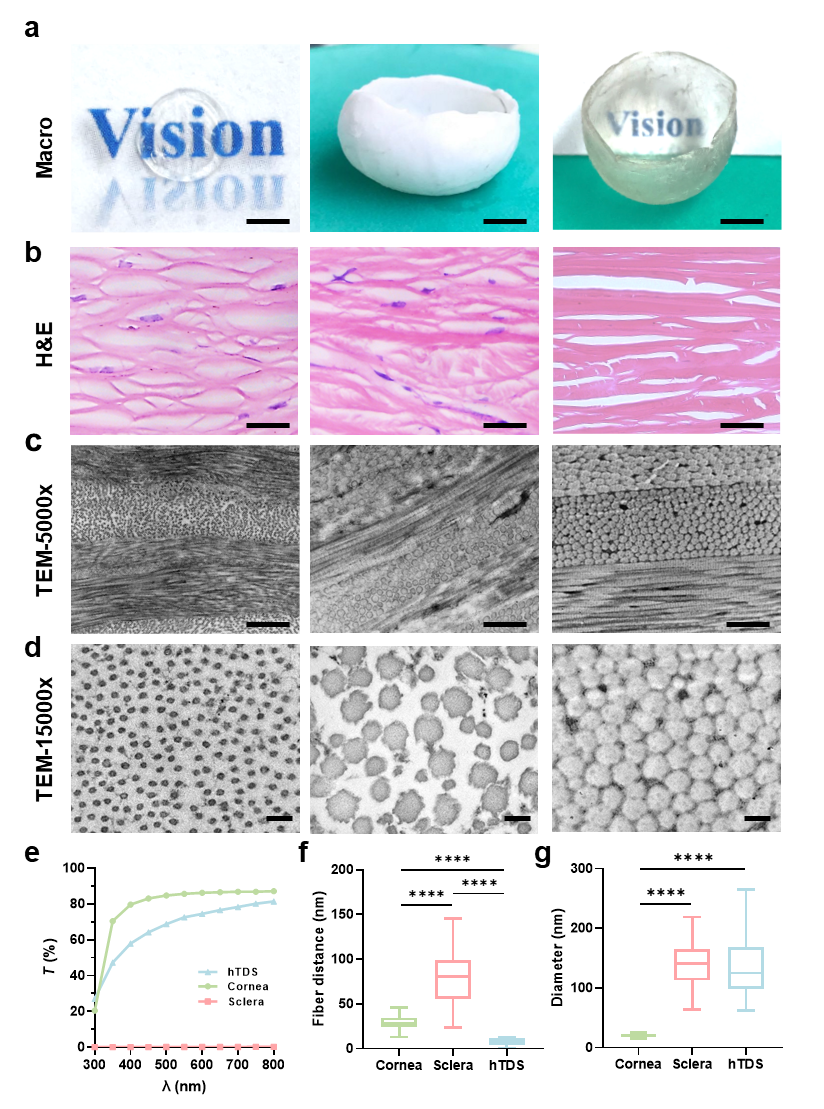


**Figure S1.** **Morphological and architectural comparison of cornea, sclera, and hTDS.** **(a)** Macroscopic appearance and transparency of cornea, sclera, and hTDS. Scale bar: 4 mm. **(b)** H&E staining images of cornea, sclera, and hTDS. Scale bar: 30 μm. **(c)**Transmission electron microscopy (TEM) images at TEM-5000×, scale bar: 500 nm, and **(d)** TEM-15000×, scale bar: 100 nm. **(e)** Light transmittance spectra, **(f)** interfibrillar distances, and **(g)** fibril diameter comparisons of cornea, sclera, and hTDS (n = 3 independent samples; ANOVA followed by Tukey’s multiple comparisons;**** adjusted *P* < 0.0001; data are presented as mean ± SD).


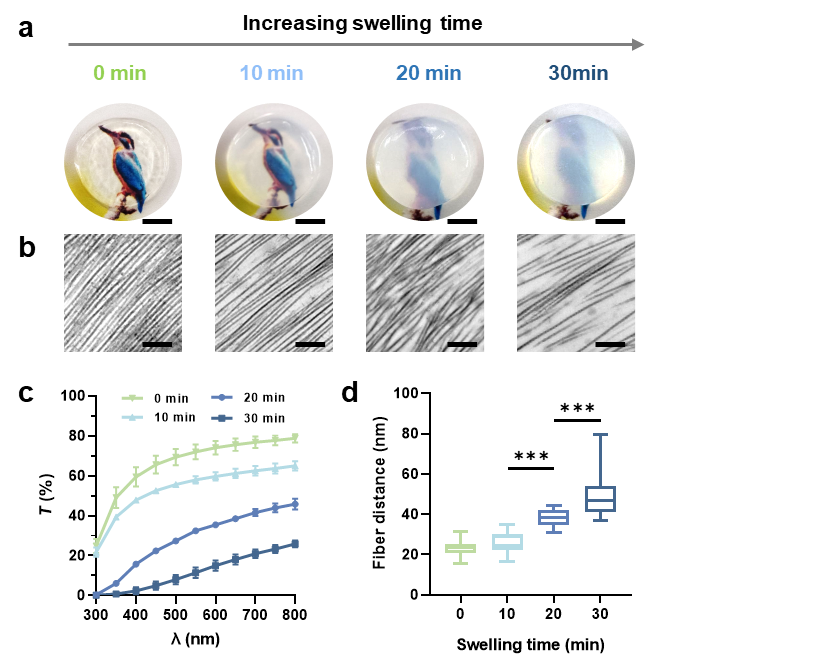


**Figure S2. Transparency and ultrastructural changes in corneal swelling. (a)** Changes in macroscopic transparency of the cornea at different swelling timepoints, showing a gradual decrease in transparency with increasing swelling time. Scale bar: 3 mm. **(b)** Representative ultrastructural images of the corneal stroma at different swelling timepoints, showing increased interfibrillar distances with increasing swelling time. Scale bar: 300 nm. **(c)** Transmittance spectra and **(d)** interfibrillar distances at different swelling timepoints (n = 3 independent samples; ANOVA followed by Tukey’s multiple comparisons;*** adjusted *P* < 0.001; data are presented as mean ± SD).

**
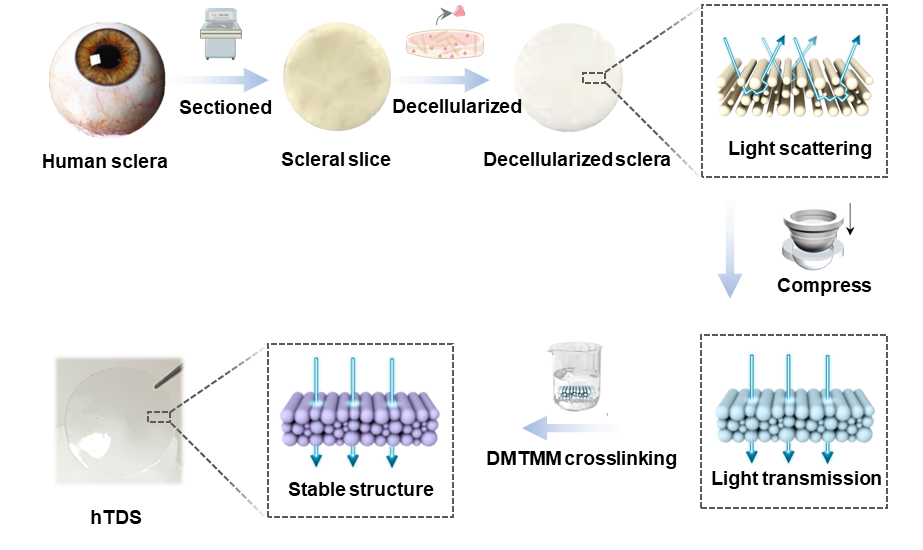
**

**Figure S3.** Schematic illustration of the hTDS fabrication.

**Figure S4.** Variation of contact ratio with packing density. To verify the underlying physics, we developed a computational framework simulating the circular boundaries of collagen fibril within a 50×50 µm² domain. Radii (0.05–0.25 µm) and positions of these objects were randomized iteratively until the cumulative area met the target packing density (30–90%), ensuring fidelity to biologically observed packing states (Figure 2e). Contact ratios were identified via pairwise Euclidean distance calculations: contacts occurred when the distance between fibril centroids was less than the sum of their radii. Dynamic memory allocation and vectorized operations optimized computational efficiency throughout the process. Results were averaged over 100 independent simulations to reduce stochastic noise. The contact ratio, defined as the proportion of fibrils engaged in ≥1 contact, was quantified to evaluate network connectivity.


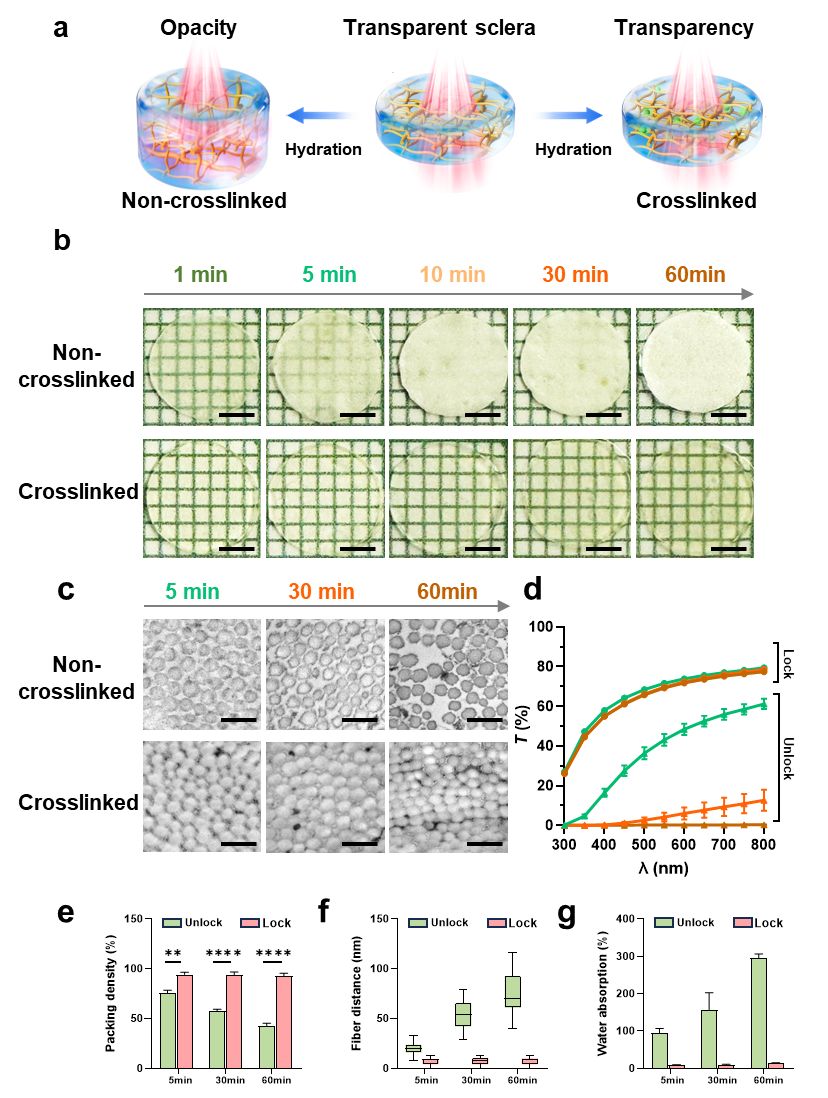


**Figure S5. The optical properties and ultrastructure of crosslinked and non-crosslinked transparentized sclera after hydration.** **(a)** Schematic diagram illustrates that the non-crosslinked transparentized sclera undergoes tissue swelling and fiber redispersion upon hydration, leading to light scattering and tissue opacity. In contrast, the dense fiber arrangement is “locked” in crosslinked sclera, ensuring refractive index homogenization and unimpeded light propagation. **(b)** Macroscopic transparency of non-crosslinked and crosslinked hTDS after hydration. Scale bars: 3 mm. **(c)** TEM images of non-crosslinked and crosslinked hTDS at different hydration time points. Scale bar: 300 nm. **(d)** Transmittance spectra, **(e)** matrix fiber packing density, **(f)** interfibrillar distances and **(g)** water absorption of non-crosslinked and crosslinked hTDS at different timepoints (crosslinked transparentized sclera is referred to as “Lock”, while non-crosslinked ones are referred to as “Unlock”; n = 3; ANOVA followed by Tukey’s multiple comparisons; ** adjusted P < 0.001,**** adjusted P < 0.0001; data are presented as mean ± SD).


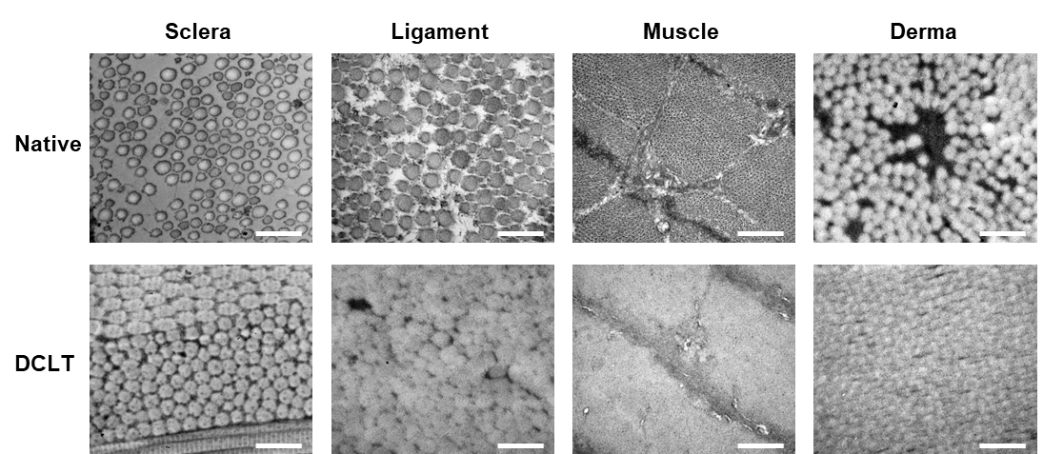


**Figure S6.** Ultrastructural comparison of matrix fibrils in native and DCLT treated sclera, ligament, muscle, and derma tissues. Scale bars: 400 nm.


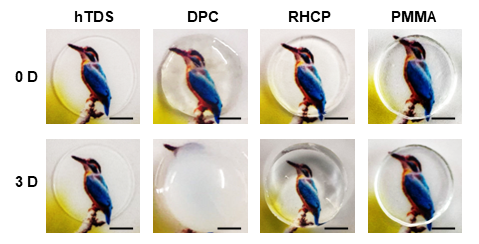


**Figure S7.** Macroscopic transparency of hTDS, decellularized porcine cornea (DPC), recombinant human collagen patches (RHCP), and poly (methyl methacrylate) (PMMA) after 3-day immersion in artificial tears. Scale bars: 3.5 mm.


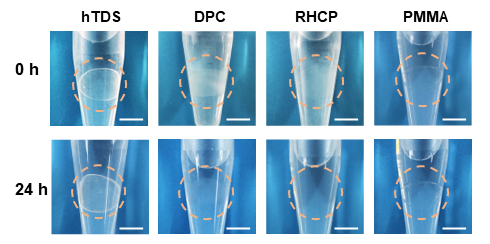


**Figure S8.** Morphology of hTDS, DPC, RHCP, and PMMA after 24 h of collagenase digestion. Scale bars: 5 mm.


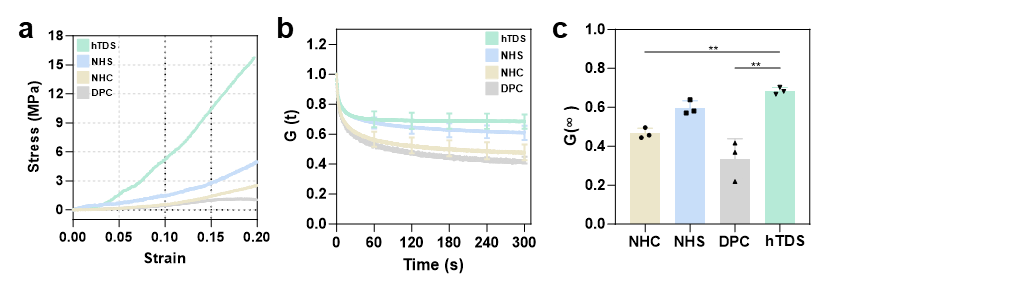


**Figure S9. Biomechanical properties of hTDS. (a)** Representative stress-strain curves (0-20% strain) of hTDS, native human sclera (NHS), native human cornea (NHC), and DPC. **(b)** Stress-relaxation behavior and **(c)** equilibrium relaxation modulus of hTDS, NHS, NHC and DPC (n = 3 independent samples; ANOVA followed by Tukey’s multiple comparisons; ** adjusted *P* < 0.01; data are presented as mean ± SD).


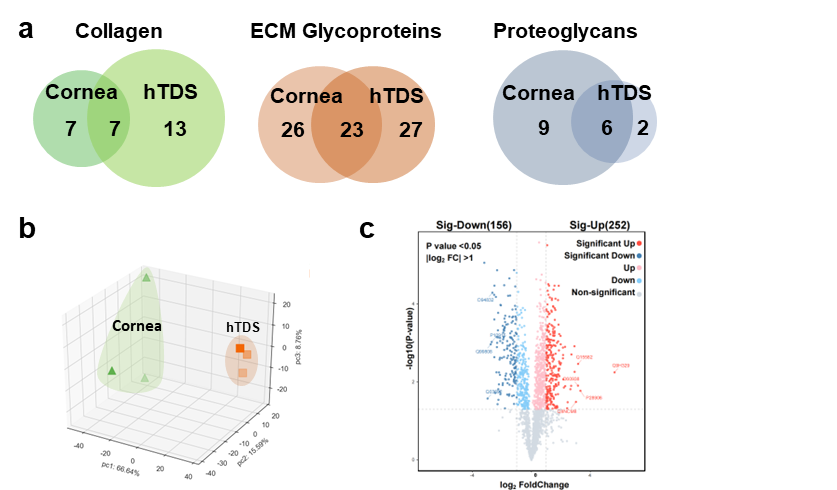


**Figure S10.** **Proteomic comparison between hTDS and human cornea. (a)** Number of core matrisome proteins detected in hTDS and human cornea. **(b)** Principal component analysis of proteomic profiles. **(c)** Volcano plot of protein expression in hTDS vs human cornea.


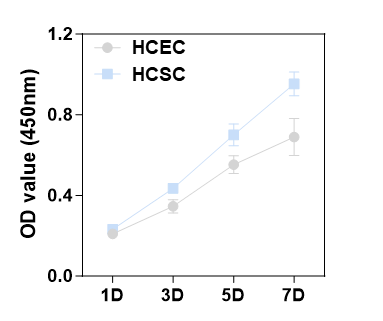


**Figure S11.** Proliferation kinetics of human corneal epithelial cells (HCECs) and stromal cells (HCSCs) by CCK8 assay in the hTDS-conditioned medium (n = 3 independent samples; data are presented as mean ± SD).


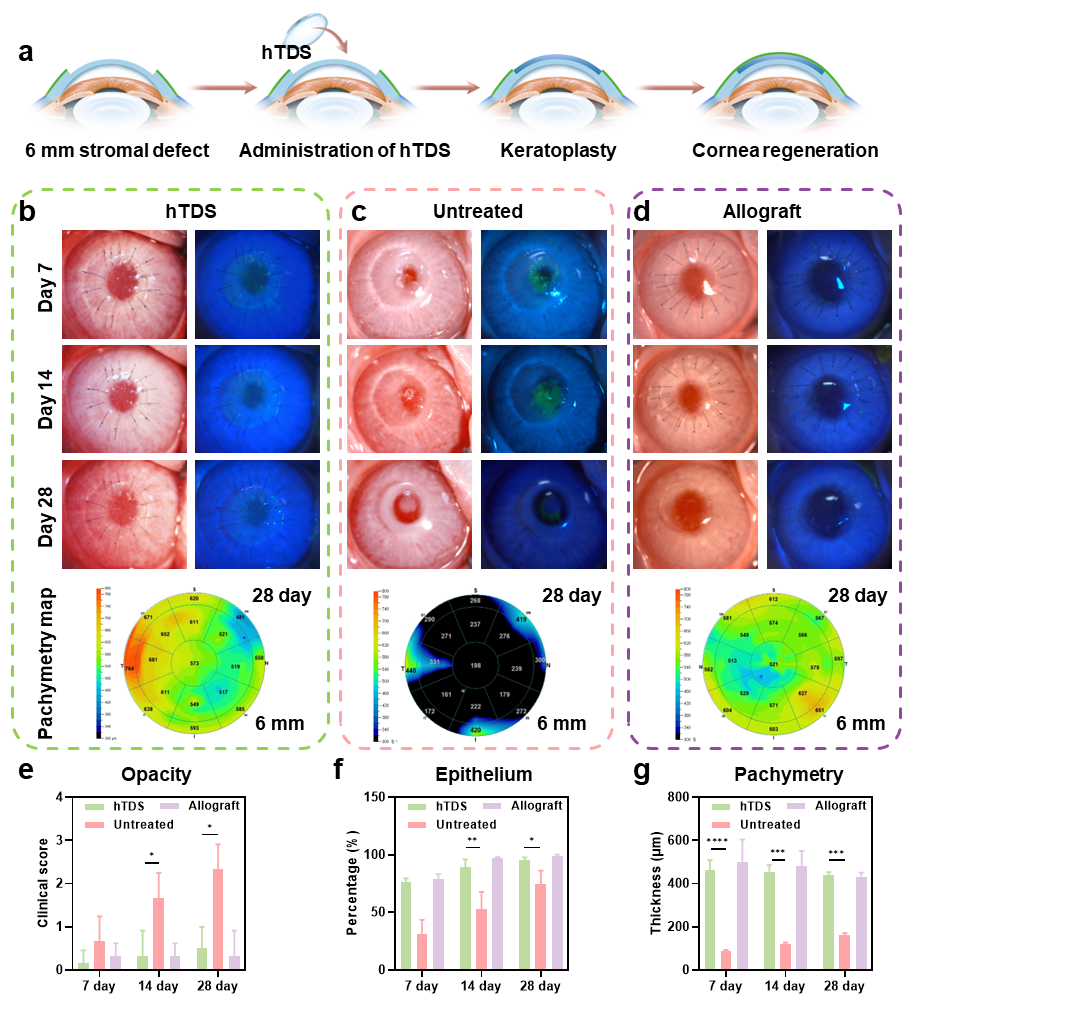


**Figure S12.** **Lamellar stromal replacement in rabbit cornea. (a)** Schematic of lamellar defect model establishment and hTDS transplantation. **(b)** Bright field, fluorescence staining and pachymetry mapping of hTDS, **(c)** untreated, and **(d)** allograft groups. **(e)** The progression of corneal opacity, **(f)** epithelial healing rate and **(g)** central corneal thickness of hTDS, untreated and allograft groups (n = 4 independent samples; ANOVA followed by Tukey’s multiple comparisons; adjusted **P* < 0.05, ** adjusted *P* < 0.01, *** adjusted *P* < 0.001; data are presented as mean ± SD).


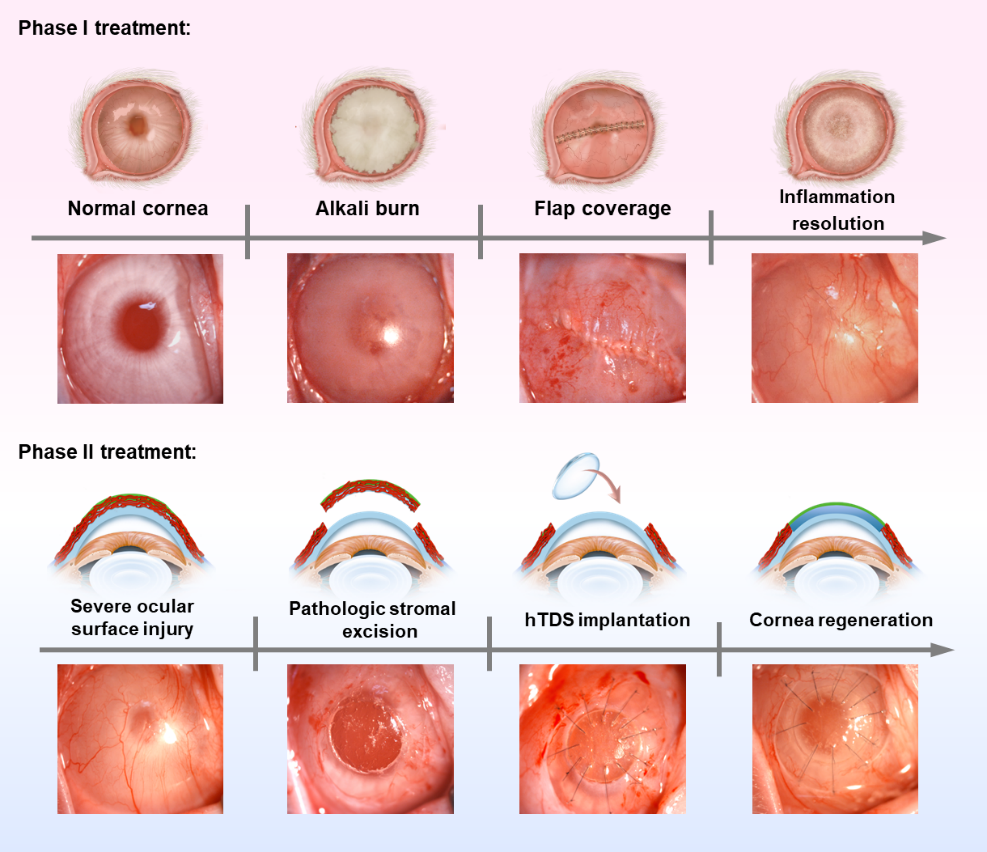


**Figure S13.** Schematic and representative images of alkali burn model establishment and staged therapeutic interventions.


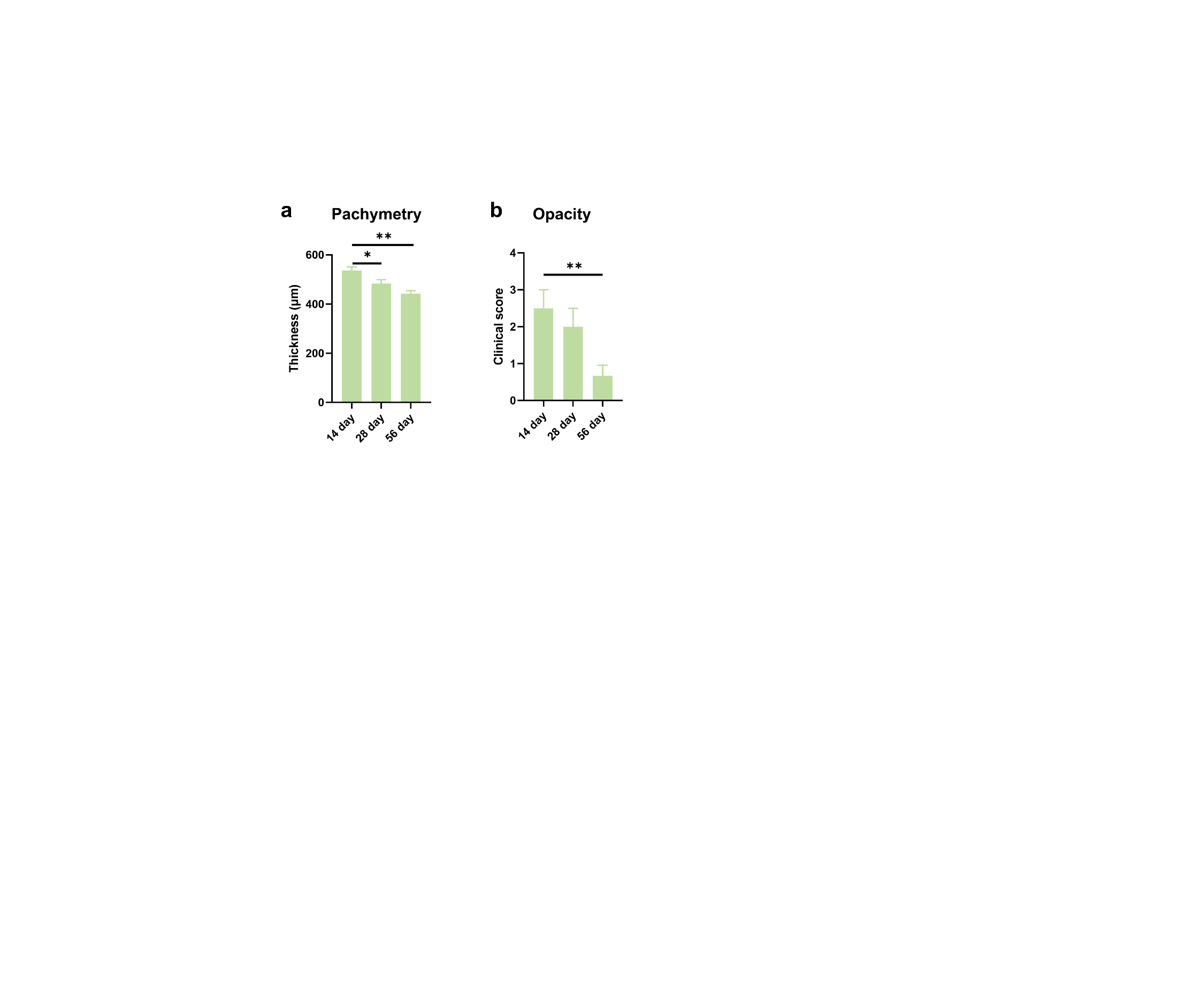


**Figure S14.** **Therapeutic outcomes in acute edematous keratoconus treated with hTDS. (a)** Central corneal thickness and **(b)** opacity score at 14, 28, and 56 days post-operation (n = 4 independent samples; ANOVA followed by Tukey’s multiple comparisons; * adjusted P < 0.05, ** adjusted P < 0.01; data are presented as mean ± SD).


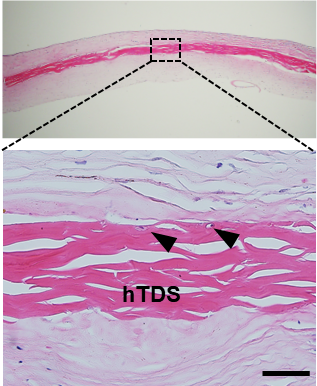


**Figure S15. H&E staining of the hTDS implanted in the stromal pocket at 56 days.** The black arrows indicate infiltrating corneal stromal cells within the graft. Scale bars: 50 μm.


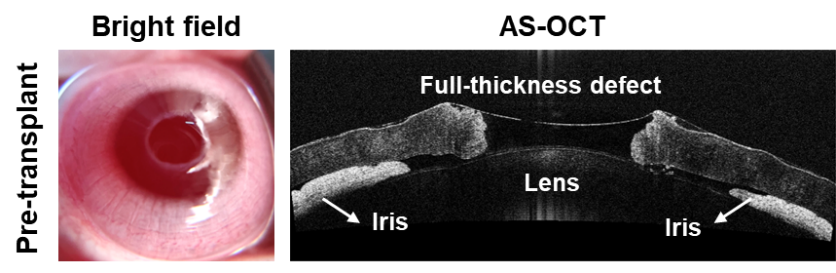


**Figure S16.** Bright-field and AS-OCT images of penetrating corneal trauma.

**
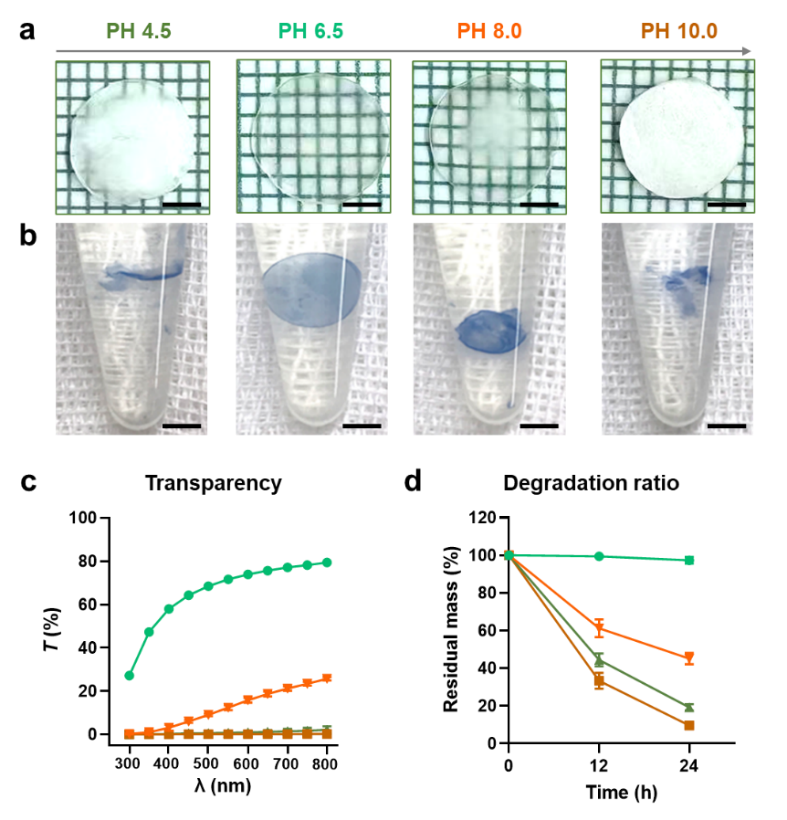
**

**Figure S17.** **Transparency and collagenase degradation of hTDS under different pH cross-linking conditions.** **(a)** Macroscopic images of hTDS treated in DMTMM solutions at pH 4.5, 6.5, 8.0, and 10.0 for 30 minutes. Scale bars: 2 mm. **(b)** Physical states of hTDS under different pH cross-linking conditions after incubation in collagenase solution. For visualization, the materials were stained with 0.4% trypan blue. Scale bars: 3 mm. **(c)** Representative transmittance spectra at the central region of the samples. **(d)** Residual mass percentage following collagenase digestion (n = 3 independent samples; data are presented as mean ± SD).

**2. Supplementary Tables**

**Table S1.** Summary of key biophysical properties and in vivo studies of cell-free corneal substitutes

| **Typical corneal substitutes** | **Subtype** | **Transparency 600 nm %** | **Epithelial healing** | **Suturability** | **Elastic modulus MPa** | **Swelling ratio %** | **In–vivo application** | **In–vivo stability** | **Sources** |
| --- | --- | --- | --- | --- | --- | --- | --- | --- | --- |
| Acellular xenogeneic scaffold | Acellular cornea | 60–80 ^[1, 2]^ | Yes ^[2]^ | Interrupted suture ^[1-7]^ | 2–10 ^[5, 8]^ | 300–600 ^[5, 7]^ | LK ^[3, 4, 6]^ | Early melting due to rejection ^[3, 4, 6, 9]^ | Pig |
|  | Acellular non–corneal tissues | 88–95 ^[10, 11]^ | No ^[12, 13]^ | Interrupted suture ^[12, 13]^ | 50–270 ^[14-16]^ | Limited swelling ^[10]^ | LK ^[13]^, IK ^[13, 17]^, PK ^[12]^ in normal cornea | Chronic inflammation and corneal melting ^[13]^ | Fish scales, swim bladder, squid mantle |
| Corneal hydrogel | Patch | 85–95 ^[18, 19]^ | Yes ^[19, 20]^ | Overlaying sutures ^[20, 21]^ | 0.2–0.5 ^[18, 19]^ | 3–10 ^[22, 23]^ | LK ^[19, 20]^, IK ^[24]^ | Postoperative thinning in LK ^[24]^ | Animal/recombinant collagen |
|  | Injectable | 80–99 ^[25, 26]^ | Yes ^[26]^ | Sutureless ^[25, 26]^ | 0.02–0.3 ^[25, 27, 28]^ | 2–20 ^[29, 30]^ | Focal defects and micro-perforations in normal cornea ^[25, 26]^ | Degradation in 2–4 weeks ^[27, 28]^ | Gelatin, animal ECM |
| Keratoprosthesis | Hard artificial cornea | >80 ^[31]^ | No ^[32]^ | Reliance on donor skirt ^[33]^ | 1500–3100 ^[34]^ | Limited swelling ^[35]^ | PK ^[32]^ | Retroprosthetic membrane in >65% patients ^[36]^ | PMMA |
|  | Soft artificial cornea | >90 ^[37, 38]^ | No ^[37]^ | Interrupted suture ^[37]^ | 0.2–5.3 ^[38, 39]^ | Limited swelling ^[37]^ | PK ^[35]^ | Stromal melting in 60% patients ^[40]^ | PHEMA, PVA |
| Human tissue grafts | Amniotic membrane | 60–70 ^[41]^ | Yes ^[42]^ | Interrupted suture ^[42]^ | 20–65 ^[43]^ | N/A | Corneal ulcers and micro-perforations ^[42, 44]^ | Prone to early dissolution ^[45, 46]^ | Human placenta |
|  | Conjunctiva | Semitransparent ^[47]^ | Yes ^[46]^ | Interrupted suture ^[46]^ | 8–10 ^[47]^ | N/A | Corneal ulcer and micro-perforations ^[48]^ | Stable white spots ^[46]^ | Human conjunctiva |
|  | Dermis | Semitransparent ^[49]^ | Yes ^[50]^ | Interrupted suture ^[51]^ | 6–6.5 ^[51]^ | N/A | IK ^[50]^ | Nebula and macula in 75% patients ^[50]^ | Human dermis |
|  | Sclera | Opaque ^[52]^ | Yes ^[53]^ | Interrupted suture ^[54]^ | 17–33 ^[55]^ | Limited swelling ^[52]^ | Eyeball rupture or perforation repair ^[53]^ | Vascular ingrowth and corneal opacity ^[56]^ | Human sclera |
| DCLT | hTDS | >80 | Yes | Interrupted suture | 11.6±3.3^a^ | 4.7±1.5 | PK, LK and IK | No graft melting and long–term transparency | Human sclera |

^a^ n = 3 independent samples; data are presented as mean ± SD

Abbreviations: ECM: Extracellular matrix, PK: Penetrating keratoplasty, LK: Lamellar keratoplasty, IK: Intrastromal keratoplasty, PMMA: Polymethyl methacrylate, PHEMA: Poly (2–hydroxyethyl methacrylate), PVA: Poly (vinyl alcohol).

**Table S2.** Antibody information

| Antibodies | Company | Item No. | Dilution |
| --- | --- | --- | --- |
| α-SMA | Abcam | ab7817 | 1:400 |
| Collagen I | Abcam | ab23446 | 1:200 |
| CK3 | Abcam | ab68260 | 1:200 |
| CD31 | Abcam | Ab9498 | 1:100 |
| Alexa Fluor 594 anti-mouse | Invitrogen | A21203 | 1:500 |
| Alexa Fluor 488 anti-mouse | Invitrogen | A21202 | 1:500 |

**Table S3.** Complex salt components of artificial tear solution

| Component | Molecular formula | Concentration |
| --- | --- | --- |
| Sodium chloride | NaCl | 90.0 mmol L^-1^ |
| Potassium chloride | KCl | 16.0 mmol L^-1^ |
| Sodium citrate | Na_3_C_6_H_5_O_7_ | 1.5 mmol L^-1^ |
| Glucose | C_6_H_12_O_6_ | 0.2 mmol L^-1^ |
| Urea | CO(NH_2_)_2_ | 1.2 mmol L^-1^ |
| Calcium chloride | CaCl_2_ | 0.5 mmol L^-1^ |
| Sodium carbonate | Na_2_CO_3_ | 12.0 mmol L^-1^ |
| Potassium hydrogen carbonate | KHCO_3_ | 3.0 mmol L^-1^ |
| Sodium phosphate dibasic | Na_2_HPO_4_ | 24.0 mmol L^-1^ |
| Human lysozyme |  | 1.8 g L^-1^ |
| Lactoferrin |  | 1.9 g L^-1^ |

**Table S4.** Clinical scoring of corneal opacity and vascularization

| Score | Opacity | Score | Vascularization |
| --- | --- | --- | --- |
| 0 | No corneal opacity | 0 | No corneal vascularization |
| 1 | 1–25% area of corneal opacity | 1 | Vessels < 1 mm axially |
| 2 | 26–50% area of corneal opacity | 2 | Vessels < 2 mm axially |
| 3 | 51–75% area of corneal opacity | 3 | Vessels < 3 mm axially |
| 4 | 76–100% area of corneal opacity | 4 | Vessels ≥ 3mm axially |

**3. References**

1. Z. Wu, Y. Zhou, N. Li, et al., “The Use of Phospholipase a(2) to Prepare Acellular Porcine Corneal Stroma as a Tissue Engineering Scaffold,” *Biomaterials* 30, no. 21 (2009): 3513.

https://doi.org/10.1016/j.biomaterials.2009.03.003

2. W. Shi, Q. Zhou, H. Gao, et al., “Protectively Decellularized Porcine Cornea Versus Human Donor Cornea for Lamellar Transplantation,” *Advanced Functional Materials* 29, no. 37 (2019): 1902491.1.

https://doi.org/10.1002/adfm.201902491

3. M. C. Zhang, X. Liu, Y. Jin, et al., “Lamellar Keratoplasty Treatment of Fungal Corneal Ulcers with Acellular Porcine Corneal Stroma,” *Am J Transplant* 15, no. 4 (2015): 1068.

https://doi.org/10.1111/ajt.13096

4. J. Zheng, X. Huang, Y. Zhang, et al., “Short-Term Results of Acellular Porcine Corneal Stroma Keratoplasty for Herpes Simplex Keratitis,” *Xenotransplantation* 26, no. 4 (2019): e12509.

https://doi.org/10.1111/xen.12509

5. H. Li, L. Zhao, F. Wang, et al., “Natural Cross-Linker-Stabilized Acellular Porcine Corneal Stroma for Lamellar Keratoplasty,” *Acta Biomaterialia* 114, (2020): 270.

https://doi.org/10.1016/j.actbio.2020.07.035

6. Y. T. Xiao, X. Y. Zhao, X. Liu, H. T. Xie, M. C. Zhang, “Ten-Year Follow-up of Lamellar Keratoplasty Treatment with Acellular Porcine Corneal Stroma: A Case Report,” *Cornea* 41, no. 5 (2022): 623.

https://doi.org/10.1097/ICO.0000000000002772

7. L. Zhao, Z. Shi, J. Wang, et al., “Natural Extracellular Matrix Scaffold-Based Hydrogel Corneal Patch with Temperature and Light-Responsiveness for Penetrating Keratoplasty and Sutureless Stromal Defect Repair,” *Adv Healthc Mater* 14, no. 10 (2025): e2402567.

https://doi.org/10.1002/adhm.202402567

8. K. Pang, L. Du, X. Wu, “A Rabbit Anterior Cornea Replacement Derived from Acellular Porcine Cornea Matrix, Epithelial Cells and Keratocytes,” *Biomaterials* 31, no. 28 (2010): 7257.

https://doi.org/10.1016/j.biomaterials.2010.05.066

9. A. Borgia, M. Airaldi, N. Lagali, et al., “Early Sterile Keratolysis Complication with Decellularized Porcine Corneal Inlay Implant: A Case Report and Cautionary Tale,” *Cornea* (2024).

https://doi.org/10.1097/ICO.0000000000003764

10. K. Tjoa, M. H. Nadhif, S. S. Utami, et al., “Mechanical, Optical, Chemical, and Biological Evaluations of Fish Scale-Derived Scaffold for Corneal Replacements: A Systematic Review,” *Int J Biol Macromol* 267, no. Pt 1 (2024): 131183.

https://doi.org/10.1016/j.ijbiomac.2024.131183

11. Y. Yin, L. Long, N. Wang, et al., “Highly Transparent and Elastic Acellular Swim Bladder with Potential Application in Cornea Implantation,” *J Mater Chem B* 13, no. 23 (2025): 6689.

https://doi.org/10.1039/d5tb00793c

12. S. C. Chen, N. Telinius, H. T. Lin, et al., “Use of Fish Scale-Derived Biocornea to Seal Full-Thickness Corneal Perforations in Pig Models,” *PLoS One* 10, no. 11 (2015): e0143511.

https://doi.org/10.1371/journal.pone.0143511

13. T. H. van Essen, C. C. Lin, A. K. Hussain, et al., “A Fish Scale-Derived Collagen Matrix as Artificial Cornea in Rats: Properties and Potential,” *Invest Ophthalmol Vis Sci* 54, no. 5 (2013): 3224.

https://doi.org/10.1167/iovs.13-11799

14. C. E. Ghezzi, B. Marelli, F. G. Omenetto, J. L. Funderburgh, D. L. Kaplan, “3d Functional Corneal Stromal Tissue Equivalent Based on Corneal Stromal Stem Cells and Multi-Layered Silk Film Architecture,” *PLoS One* 12, no. 1 (2017): e0169504.

https://doi.org/10.1371/journal.pone.0169504

15. B. Aghaei-Ghareh-Bolagh, J. Guan, Y. Wang, et al., “Optically Robust, Highly Permeable and Elastic Protein Films That Support Dual Cornea Cell Types,” *Biomaterials* 188, (2019): 50.

https://doi.org/10.1016/j.biomaterials.2018.10.006

16. K. Long, Y. Liu, W. Li, et al., “Improving the Mechanical Properties of Collagen-Based Membranes Using Silk Fibroin for Corneal Tissue Engineering,” *J Biomed Mater Res A* 103, no. 3 (2015): 1159.

https://doi.org/10.1002/jbm.a.35268

17. H. Kang, Y. Han, M. Jin, et al., “Decellularized Squid Mantle Scaffolds as Tissue-Engineered Corneal Stroma for Promoting Corneal Regeneration,” *Bioeng Transl Med* 8, no. 4 (2023): e10531.

https://doi.org/10.1002/btm2.10531

18. E. O. Osidak, A. Y. Andreev, S. E. Avetisov, et al., “Corneal Stroma Regeneration with Collagen-Based Hydrogel as an Artificial Stroma Equivalent: A Comprehensive in Vivo Study,” *Polymers (Basel)* 14, no. 19 (2022).

https://doi.org/10.3390/polym14194017

19. P. Fagerholm, N. S. Lagali, J. A. Ong, et al., “Stable Corneal Regeneration Four Years after Implantation of a Cell-Free Recombinant Human Collagen Scaffold,” *Biomaterials* 35, no. 8 (2014): 2420.

https://doi.org/10.1016/j.biomaterials.2013.11.079

20. P. Fagerholm, N. S. Lagali, K. Merrett, et al., “A Biosynthetic Alternative to Human Donor Tissue for Inducing Corneal Regeneration: 24-Month Follow-up of a Phase 1 Clinical Study,” *Sci Transl Med* 2, no. 46 (2010): 46ra61.

https://doi.org/10.1126/scitranslmed.3001022

21. M. Xeroudaki, M. Thangavelu, A. Lennikov, et al., “A Porous Collagen-Based Hydrogel and Implantation Method for Corneal Stromal Regeneration and Sustained Local Drug Delivery,” *Scientific Reports* 10, no. 1 (2020): 16936.

https://doi.org/10.1038/s41598-020-73730-9

22. L. Zhao, Z. Shi, X. Qi, et al., “Corneal Stromal Structure Replicating Humanized Hydrogel Patch for Sutureless Repair of Deep Anterior-Corneal Defect,” *Biomaterials* 313, (2025): 122754.

https://doi.org/10.1016/j.biomaterials.2024.122754

23. M. Lei, S. Zhang, H. Zhou, et al., “Electrical Signal Initiates Kinetic Assembly of Collagen to Construct Optically Transparent and Geometry Customized Artificial Cornea Substitutes,” *ACS Nano* 16, no. 7 (2022): 10632.

https://doi.org/10.1021/acsnano.2c02291

24. M. Rafat, M. Jabbarvand, N. Sharma, et al., “Bioengineered Corneal Tissue for Minimally Invasive Vision Restoration in Advanced Keratoconus in Two Clinical Cohorts,” *Nature Biotechnology* 41, no. 1 (2023): 70.

https://doi.org/10.1038/s41587-022-01408-w

25. C. D. McTiernan, F. C. Simpson, M. Haagdorens, et al., “Liqd Cornea: Pro-Regeneration Collagen Mimetics as Patches and Alternatives to Corneal Transplantation,” *Science Advances* 6, no. 25 (2020).

https://doi.org/10.1126/sciadv.aba2187

26. L. Zhao, Z. Shi, X. L. Sun, et al., “Natural Dual-Crosslinking Bioadhesive Hydrogel for Corneal Regeneration in Large-Size Defects,” *Advanced Healthcare Materials* 11, no. 21 (2022).

https://doi.org/10.1002/adhm.202201576

27. E. S. Sani, A. Kheirkhah, D. Rana, et al., “Sutureless Repair of Corneal Injuries Using Naturally Derived Bioadhesive Hydrogels,” *Science Advances* 5, no. 3 (2019).

https://doi.org/10.1126/sciadv.aav1281

28. Y. A. Qian, K. J. Xu, L. L. Shen, et al., “Dopamine-Based High-Transparent Hydrogel as Bioadhesive for Sutureless Ocular Tissue Repair,” *Advanced Functional Materials* 33, no. 49 (2023).

https://doi.org/10.1002/adfm.202300707

29. H. Kim, J. H. Jang, W. N. Han, et al., “Extracellular Matrix-Based Sticky Sealants for Scar-Free Corneal Tissue Reconstruction,” *Biomaterials* 292, (2023).

https://doi.org/10.1016/j.biomaterials.2022.121941

30. X. R. Shen, S. Q. Li, X. Zhao, et al., “Dual-Crosslinked Regenerative Hydrogel for Sutureless Long-Term Repair of Corneal Defect,” *Bioactive Materials* 20, (2023): 434.

https://doi.org/10.1016/j.bioactmat.2022.06.006

31. M. Gonzalez-Andrades, R. Sharifi, M. M. Islam, et al., “Improving the Practicality and Safety of Artificial Corneas: Pre-Assembly and Gamma-Rays Sterilization of the Boston Keratoprosthesis,” *Ocular Surface* 16, no. 3 (2018): 322.

https://doi.org/10.1016/j.jtos.2018.04.002

32. M. Moshirfar, J. J. Moody, M. R. Barke, et al., “The Historical Development and an Overview of Contemporary Keratoprostheses,” *Surv Ophthalmol* 67, no. 4 (2022): 1175.

https://doi.org/10.1016/j.survophthal.2022.01.005

33. F. L. and E. J. Hollick 2023. “Artificial Cornea Transplantation.” in StatPearls [Internet], Treasure Island (FL): StatPearls Publishing. PMID: 33760451.

34. F. Van Loock, N. A. Fleck, “Deformation and Failure Maps for Pmma in Uniaxial Tension,” *Polymer* 148, (2018): 259.

https://doi.org/10.1016/j.polymer.2018.06.027

35. G. Holland, A. Pandit, L. Sanchez-Abella, et al., “Artificial Cornea: Past, Current, and Future Directions,” *Front Med (Lausanne)* 8, (2021): 770780.

https://doi.org/10.3389/fmed.2021.770780

36. J. Park, P. Phrueksaudomchai, M. S. Cortina, “Retroprosthetic Membrane: A Complication of Keratoprosthesis with Broad Consequences,” *Ocular Surface* 18, no. 4 (2020): 893.

https://doi.org/10.1016/j.jtos.2020.09.004

37. J. Pan, W. Zhang, J. Zhu, et al., “Arrested Phase Separation Enables High-Performance Keratoprostheses,” *Advanced Materials* 35, no. 16 (2023): e2207750.

https://doi.org/10.1002/adma.202207750

38. H. Jiang, Y. Zuo, L. Zhang, et al., “Property-Based Design: Optimization and Characterization of Polyvinyl Alcohol (Pva) Hydrogel and Pva-Matrix Composite for Artificial Cornea,” *J Mater Sci Mater Med* 25, no. 3 (2014): 941.

https://doi.org/10.1007/s10856-013-5121-0

39. G. Trujillo-de Santiago, R. Sharifi, K. Yue, et al., “Ocular Adhesives: Design, Chemistry, Crosslinking Mechanisms, and Applications,” *Biomaterials* 197, (2019): 345.

https://doi.org/10.1016/j.biomaterials.2019.01.011

40. N. Jirásková, P. Rozsival, M. Burova, M. Kalfertova, “Alphacor Artificial Cornea: Clinical Outcome,” *Eye* 25, no. 9 (2011): 1138.

https://doi.org/10.1038/eye.2011.122

41. T. Deihim, G. Yazdanpanah, H. Niknejad, “Different Light Transmittance of Placental and Reflected Regions of Human Amniotic Membrane That Could Be Crucial for Corneal Tissue Engineering,” *Cornea* 35, no. 7 (2016): 997.

https://doi.org/10.1097/ico.0000000000000867

42. K. Krysik, D. Dobrowolski, E. Wylegala, A. Lyssek-Boron, “Amniotic Membrane as a Main Component in Treatments Supporting Healing and Patch Grafts in Corneal Melting and Perforations,” *Journal of Ophthalmology* 2020, (2020).

https://doi.org/10.1155/2020/4238919

43. R. S. Chuck, J. M. Graff, M. R. Bryant, P. M. Sweet, “Biomechanical Characterization of Human Amniotic Membrane Preparations for Ocular Surface Reconstruction,” *Ophthalmic Research* 36, no. 6 (2004): 341.

https://doi.org/10.1159/000081637

44. D. Meller, M. Pauklin, H. Thomasen, H. Westekemper, K. P. Steuhl, “Amniotic Membrane Transplantation in the Human Eye,” *Deutsches Arzteblatt International* 108, no. 14 (2011): 243.

https://doi.org/10.3238/arztebl.2011.0243

45. H. Tsujita, A. B. Brennan, C. E. Plummer, et al., “An

Model for Suture-Less Amniotic Membrane Transplantation with a Chemically Defined Bioadhesive,” *Current Eye Research* 37, no. 5 (2012): 372.

https://doi.org/10.3109/02713683.2012.663853

46. Y. L. Du, J. S. Wang, W. Geng, et al., “Amniotic Membrane Transplantation Combined with Conjunctival Flap Covering Surgery for the Treatment of Corneal Perforations in Fungal Keratitis,” *Heliyon* 9, no. 12 (2023): e22693.

https://doi.org/10.1016/j.heliyon.2023.e22693

47. L. Zhao, Y. Jia, C. Zhao, et al., “Ocular Surface Repair Using Decellularized Porcine Conjunctiva,” *Acta Biomaterialia* 101, (2020): 344.

https://doi.org/10.1016/j.actbio.2019.11.006

48. N. S. Shekhawat, B. Kaur, A. Edalati, M. Abousy, A. O. Eghrari, “Tenon Patch Graft with Vascularized Conjunctival Flap for Management of Corneal Perforation,” *Cornea* 41, no. 11 (2022): 1465.

https://doi.org/10.1097/ICO.0000000000003068

49. Z. Y. Liu, J. Ji, J. Zhang, et al., “Corneal Reinforcement Using an Acellular Dermal Matrix for an Analysis of Biocompatibility, Mechanical Properties, and Transparency,” *Acta Biomaterialia* 8, no. 9 (2012): 3326.

https://doi.org/10.1016/j.actbio.2012.05.004

50. X. D. Jiang, Y. X. Wang, W. Q. Qiu, et al., “Corneal Stromal Transplantation with Human-Derived Acellular Dermal Matrix for Pellucid Marginal Corneal Degeneration: A Nonrandomized Clinical Trial,” *Transplantation* 103, no. 6 (2019): E172.

https://doi.org/10.1097/Tp.0000000000002681

51. Y. X. Wang, J. H. Ma, X. D. Jiang, et al., “Development of Transparent Acellular Dermal Matrix as Tissue-Engineered Stroma Substitute for Central Lamellar Keratoplasty,” *Investigative Ophthalmology & Visual Science* 61, no. 1 (2020).

https://doi.org/ 10.1167/iovs.61.1.5

52. C. Boote, I. A. Sigal, R. Grytz, et al., “Scleral Structure and Biomechanics,” *Progress in Retinal and Eye Research* 74, (2020).

https://doi.org/10.1016/j.preteyeres.2019.100773

53. U. Karaca, G. Usta, “The Usability of Lamellar Scleral Autograft in Ocular Perforation Treatment,” *Int Ophthalmol* 42, no. 2 (2022): 377.

https://doi.org/10.1007/s10792-021-01922-x

54. P. Prasher, “Use of an Autologous Lamellar Scleral Graft to Repair a Corneal Perforation,” *International Ophthalmology* 34, no. 4 (2014): 957.

https://doi.org/10.1007/s10792-013-9883-7

55. B. Geraghty, S. W. Jones, P. Rama, R. Akhtar, A. Elsheikh, “Age-Related Variations in the Biomechanical Properties of Human Sclera,” *Journal of the Mechanical Behavior of Biomedical Materials* 16, (2012): 181.

https://doi.org/10.1016/j.jmbbm.2012.10.011

56. J. E. Winkelman, “Optical Behavior of Sclera Transplanted into Cornea,” *Am J Ophthalmol* 34, no. 10 (1951): 1379.

https://doi.org/10.1016/0002-9394(51)90477-1
